# PP‐1β and PP‐2Aα modulate cAMP response element‐binding protein (CREB) functions in aging control and stress response through de‐regulation of αB‐crystallin gene and p300‐p53 signaling axis

**DOI:** 10.1111/acel.13458

**Published:** 2021-08-23

**Authors:** Ling Wang, Lan Zhang, Xiao‐Dong Gong, Jia‐Ling Fu, Yu‐Wen Gan, Min Hou, Qian Nie, Jia‐Wen Xiang, Yuan Xiao, Yan Wang, Shu‐Yu Zheng, Lan Yang, Huimin Chen, Meng‐Qing Xiang, Yizhi Liu, David Wan‐Cheng Li

**Affiliations:** ^1^ The State Key Laboratory of Ophthalmology Zhongshan Ophthalmic Center Sun Yat‐sen University Guangzhou China

**Keywords:** aging, apoptosis, Bak, Bax, cataract, oxidative stress, P300, p53, Pcaf, PP‐1β; PP‐2Aα; CREB

## Abstract

The function of the transcription factor, cAMP response element‐binding protein (CREB), is activated through S133 phosphorylation by PKA and others. Regarding its inactivation, it is not well defined. cAMP response element‐binding protein plays an essential role in promoting cell proliferation, neuronal survival and the synaptic plasticity associated with long‐term memory. Our recent studies have shown that CREB is an important player in mediating stress response. Here, we have demonstrated that CREB regulates aging process through suppression of αB‐crystallin and activation of the p300‐p53‐Bak/Bax signaling axis. First, we determined that two specific protein phosphatases, PP‐1β and PP‐2Aα, can inactivate CREB through S133 dephosphorylation. Subsequently, we demonstrated that cells expressing the S133A‐CREB, a mutant mimicking constant dephosphorylation at S133, suppress CREB functions in aging control and stress response. Mechanistically, S133A‐CREB not only significantly suppresses CREB control of αB‐crystallin gene, but also represses CREB‐mediated activation of p53 acetylation and downstream Bak/Bax genes. cAMP response element‐binding protein suppression of αB‐crystallin and its activation of p53 acetylation are major molecular events observed in human cataractous lenses of different age groups. Together, our results demonstrate that PP‐1β and PP‐2Aα modulate CREB functions in aging control and stress response through de‐regulation of αB‐crystallin gene and p300‐p53‐Bax/Bak signaling axis, which regulates human cataractogenesis in the aging lens.

## Graphical abstract

This figure illustrates that the PP‐1β and PP‐2Aα modulate CREB functions in aging control and stress response through suppression of alphaB‐crystallin and activation of the p300‐p53‐Bax/Bak signaling axis in normal and cataractous lenses.

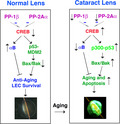

**Abbreviations**
CREB: cAMP response element‐binding protein
DMEM: Dulbecco's modified eagle medium
GO: glucose oxidase
JB6: mouse skin epithelial cells
OA: okadaic acid
PAGE: polyacrylamide gel electrophoresis
PBS: phosphate‐buffered saline
PKA: protein kinase A
RB: retina blastoma protein
SDS: sodium dodecylsulfate
TBS: tris‐buffered saline
TBS‐T: tris‐buffered saline with tween‐20
αB: αB‐crystallin
αTN4‐1: mouse lens epithelial cells

## 1 INTRODUCTION

Protein phosphorylation/dephosphorylation is one of the most important post‐translational modifications, modulating functions of more than one‐third of total eukaryote proteins, and thus participates in control of various physiological processes such as gene expression and regulation, DNA replication and damage response, cell proliferation, growth and differentiation, cell transformation, and apoptosis (Cohen, 1989; Hunter, 1995; Hunter & Karin, 1992; Moorhead et al., 2007; Mumby & Walter, 1993; Olsen et al., 2006).

It is well established that protein phosphorylation/dephosphorylation is also implicated in control of cell senescence and organism aging. Through regulating functions of the various signaling transducers and transcription factors, protein phosphorylation/dephosphorylation plays essential roles in the control of aging (Hart, 2019; Kang et al., 2013; Matsuoka et al., 2007; Pan & Finkel, 2017). The tumor suppressors, the retina blastoma protein (RB) and p53, are important players and have been shown to control two major aging pathways (Campisi, 2005; Miura et al., 2004; Pelicci, 2004; Sperka et al., 2012; Stewart & Weinberg, 2006). Their functions are highly regulated by various kinases. The cyclin‐dependent kinases phosphorylate RB to free members of E2F and thus drive cell cycle progression or run into premature senescence (Kim & Sharpless, 2006; Sharpless & DePinho, 2004). For p53, 17 serine/threonine phosphorylation residues have been identified to implicate its stability and activation status (Bode & Dong, 2004; Kruse & Gu, 2009). In this regard, we have previously demonstrated that both PP‐1 and PP‐2A can dephosphorylate p53 to modulate its transcriptional and pro‐apoptotic activities (Li et al., 2006; Qin et al., 2008). Here, we show that PP‐1 and PP‐2A can modulate the functions of the cAMP response element‐binding protein (CREB), a major transcription factor in brain functions (Mayr & Montminy, 2001) and also an important mediator of stress response (Wang et al., 2020).

cAMP response element‐binding protein mediates the regulation of the cAMP response genes by binding as a dimer to a conserved cAMP response element (CRE): TGACGTCA (Comb et al., 1986; Montminy et al., 1986). During its activation, the G‐protein‐coupled receptors induce accumulation of cAMP, which activates PKA to phosphorylate CREB at S133 (Hagiwara et al., 1993) and promotes recruitment of the transcription co‐activator CBP and its paralogue p300 (Arany et al., 1994; Chrivia et al., 1993; Del et al., 2019; Ebrahimi et al., 2019). The activated CREB is inactivated through S133 dephosphorylation. However, the exact phosphatases mediating CREB inactivation have not been well defined.

The most prominent function of CREB is its implication in the synaptic plasticity associated with long‐term memory (Altarejos & Montminy, 2011; Bartsch et al., 1998; Del et al., 2019). Disruption of CREB (both α‐ and δ‐isoforms) in mice causes defects in long‐term potentiation and long‐term memory (Bourtchuladze et al., 1994). On the contrary, expression of the dominant‐active CREB polypeptide accelerates the learning process (Yin et al., 1994, 1995). cAMP response element‐binding protein also promotes growth‐factor‐dependent survival of both sympathetic and cerebellar neurons (Bonni et al., 1999; Riccio et al., 1999).

In contrast to the earlier study, our recent studies have shown that CREB plays an important role in mediating stress response (Wang et al., 2020). By suppressing expression of αB‐crystallin in lens epithelial cells, CREB promotes oxidative stress‐induced apoptosis followed by cataractogenesis (Li et al., 1995; Li & Spector, 1996; Wang et al., 2020).

Cataract is an aging disease that in most cases is derived from aging process or environmental stress induction (Cogan, 1973) besides genetic mutations (Shiels & Hejtmancik, 2019). Over two centuries of studies have revealed that many different factors can induce cataractogenesis (Bloemendal, 1991; Lim et al., 2020; Schey et al., 2020; Shu & Lovicu, 2017; Wormstone et al., 2020). Mechanistically, we have previously demonstrated that stress‐induced apoptosis is a common cellular basis for non‐congenital cataractogenesis (Li et al., 1995; Li & Spector, 1996).

In the present study, we demonstrate that CREB regulates aging process through suppression of αB‐crystallin and activation of the p300‐p53‐Bak/Bax signaling axis. First, we determined that two specific protein phosphatases, PP‐1β and PP‐2Aα, can dephosphorylate CREB at S133 to modulate its functions. Subsequently, we showed that lens epithelial cells expressing the S133A‐CREB, a mutant mimicking constant dephosphorylation of CREB at S133, suppress the ability of wild‐type CREB in mediating aging control and stress response. Mechanistically, S133A‐CREB not only significantly de‐regulates CREB suppression of αB‐crystallin but also represses CREB‐mediated activation of p300‐p53 signaling axis. In the S133A‐CREB mice generated through CRISPR/Cas9 technology, we found that αB‐crystallin expression is greatly upregulated than in wild‐type adult mice. Moreover, PP‐1β and PP‐2Aα overexpression can negatively regulate CREB and its downstream target, αB. Furthermore, from transparent lenses to cataractous lenses of the 60s age group, we observed that the catalytic subunits of PP‐1β and PP‐2Aα are downregulated to enhance CREB functions. Consistently, CREB is significantly upregulated from transparent lenses to cataractous lenses of the same age group. In contrast, αB‐crystallin, the CREB target, an important anti‐apoptotic regulator (Andley et al., 2000; Mao et al., 2004; Martin et al., 1997; Mehlen et al., 1995; Ousman et al., 2007; Sharma, 2016), and also an anti‐aging regulator (Brady et al., 2001; Lim et al., 2017), is greatly downregulated. Therefore, CREB suppression of αB‐crystallin is closely associated with human lens aging and pathogenesis. In addition, p53 is downregulated from normal lens to cataractous lenses of the 60s age group; however, the acetyltransferase, Pcaf for p53 and p53 acetylation at K382 are significantly upregulated of the same age group. Thus, CREB‐regulated activation of p300‐p53 signaling axis is also linked to human lens aging and cataract formation. Together, our results demonstrate that dephosphorylation by PP‐1β and PP‐2Aα modulates CREB functions in aging control and stress response through de‐regulation of αB‐crystallin gene and p300‐p53‐Bax/Bak signaling axis, which regulates human cataractogenesis in the aging lenses.

## 2 RESULTS

### 2.1 Inhibition of protein phosphatases, PP‐1 and PP‐2A, induces dose‐dependent hyperphosphorylation of CREB at S133

**FIGURE 1:**
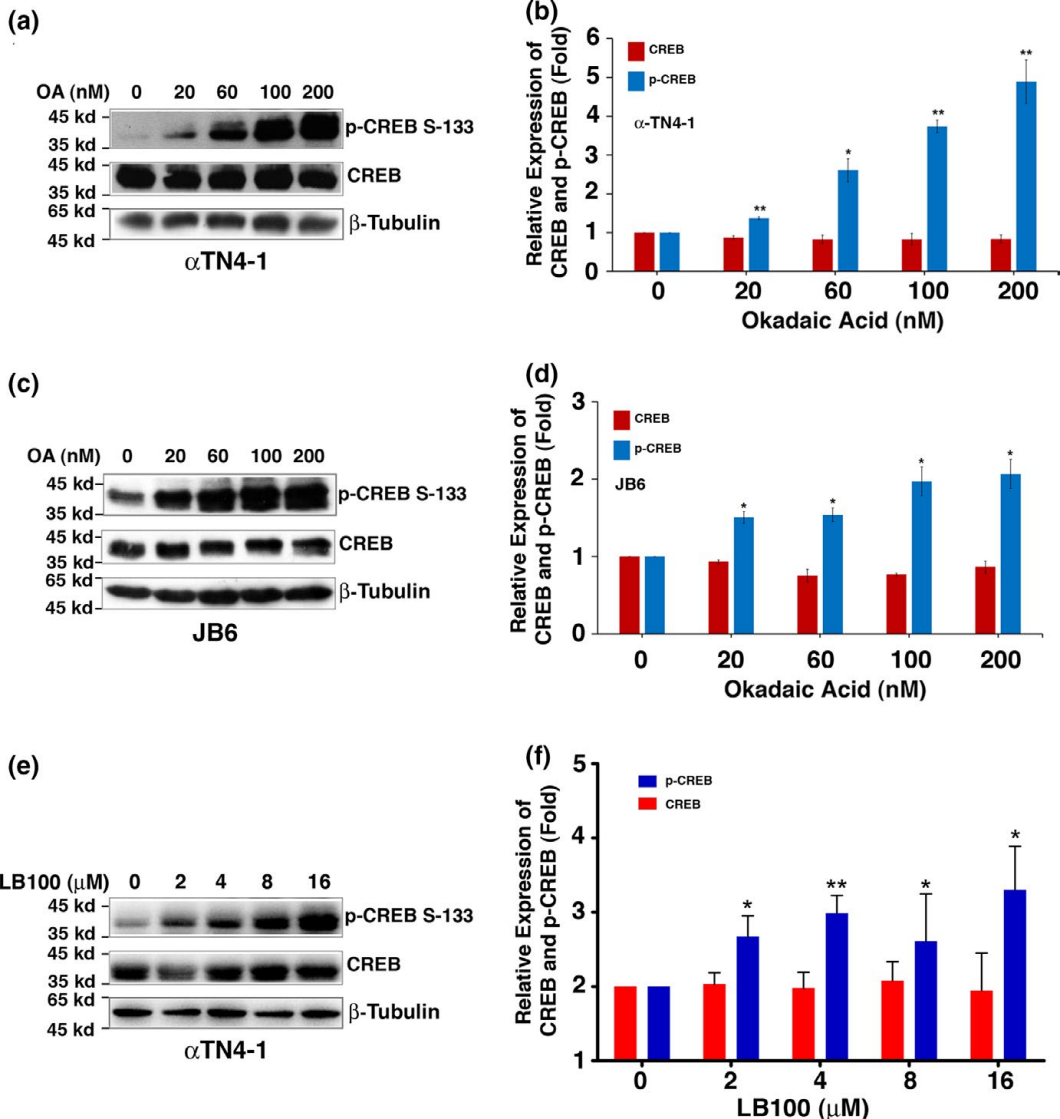
Inhibition of PP‐1 and PP‐2A by okadaic acid (OA) or PP2A inhibitor LB100 induces hyperphosphorylation of CREB at S133. Inhibition of PP‐1 and PP‐2A by okadaic acid (OA) in mouse lens epithelial cells (αTN4‐1, a & b) or mouse skin epithelial cells (JB6, c & d) induces hyperphosphorylation of CREB at S133. Both types of cells were grown to 95% confluence, then pretreated with 0.1% DMSO (lane 1) or with 20–200 nM okadaic acid as indicated for 3 h. The expression levels of CREB, p‐CREB at S133 and β‐tubulin were determined by Western blot analysis (WB). Notice that inhibition of PP‐2A (20 nM OA) or both PP‐1 and PP‐2A (20–200 nM OA) as previously determined (Li et al., 2006.) induces hyperphosphorylation of CREB at S133 in both lens epithelial cells (αTN4‐1 cells, a & b) and mouse skin epithelial cells (JB6 cells, c & d). Furthermore, LB100 a more specific inhibitor for PP‐2A at the concentrations of 2–16 μM also induced dose‐dependent hyperphosphorylation of CREB at S133 in mouse lens epithelial cells, αTN4‐1 (e & f). These results suggest that both PP‐1 and PP‐2A seem to be involved in dephosphorylation of CREB at S133. The *p*‐value was calculated by comparing the pixel density value from each OA/LB100‐treated sample with that from the mock‐treated sample. *p < 0.05, **p < 0.01

To identify the specific isoforms of phosphatases that dephosphorylate CREB at S133, we first treated the mouse lens epithelial cells (MLECs), αTN4‐1 with okadaic acid (OA), a potent inhibitor that has been shown to inhibit PP‐2A activity at low concentration (<20 nM) but both PP‐2A and PP‐1 at higher concentrations (>20 nM) (Fernandez et al., 2002; Li et al., 2006; Qin et al., 2008; Xiao et al., 2010). As shown in Figure 1a,b, OA at concentrations from 20 to 200 nM caused a dose‐dependent increase in the phosphorylation level of CREB at S133, such result suggests that both PP‐1 and PP‐2A may dephosphorylate CREB at S133 in mouse lens epithelial cells. To determine whether this is true in other cells, we also treated mouse skin epithelial cells, JB6, with the same concentration gradients. As shown in Figure 1c,d, although JB6 cells have a higher background of CREB phosphorylation at S133, treatment of these cells with 20–200 nM OA also caused similar pattern of CREB hyperphosphorylation at S133, further indicating the involvement of PP‐1 and PP‐2A in the dephosphorylation of CREB S133 in both lens and non‐lens cells. To further show that PP‐2A is implicated in control of CREB S133 dephosphorylation, we treated αTN4‐1 cells with a more specific PP‐2A inhibitor, LB100 (Wang, Lou, et al., 2019; Wang, Friedrich, et al., 2019). As shown in Figure 1e,f, LB100 at concentrations of 2–16 μM also induces dose‐dependent CREB phosphorylation, thus confirming the role of PP‐2A in this process.

### 2.2 Silence of PP‐1β and PP‐2Aα leads to hyperphosphorylation of CREB at S133 in mouse lens epithelial cells

**FIGURE 2:**
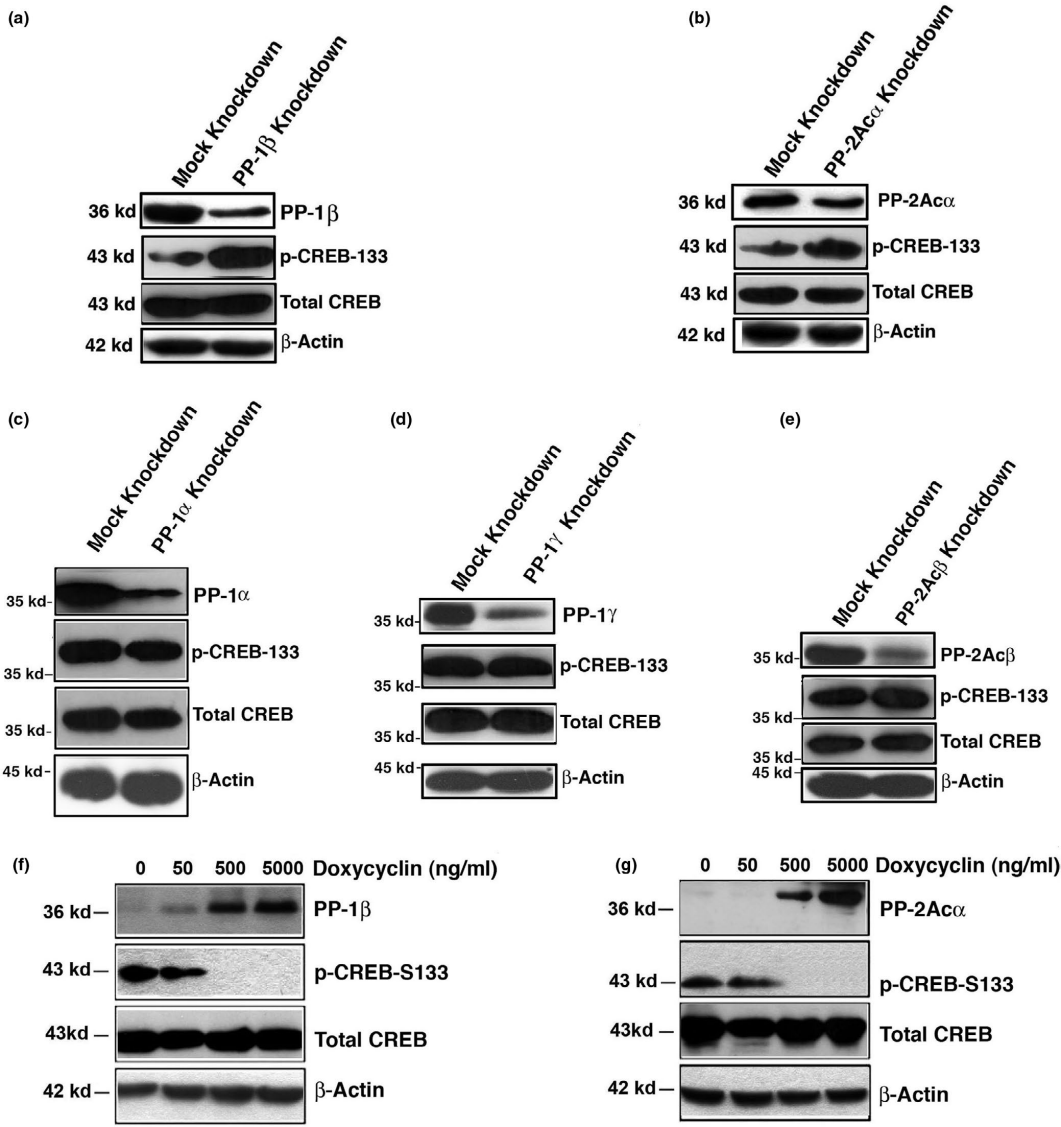
Changes in PP‐1β or PP‐2Aα levels are closely linked to phosphorylation status of CREB. (a & b) Silence of PP‐1β (a) or PP‐2Acα (b) by specific shRNAs enhances hyperphosphorylation of CREB at S133 in αTN4‐1 cells. (c, d, & e) Silence of PP‐1α (c), PP1γ (d), or PP‐2Acβ (e) by specific shRNAs almost have no effect on phosphorylation status of CREB at S133 in αTN4‐1 cells. One stable clone from each catalytic subunit silence group was selected for comparison with the mock knockdown. The different silence constructs were obtained from Open Biosystems Inc. and amplified. The different constructs were then transfected into αTN4‐1 cells, and stable clones expressing shRNA for each catalytic subunit of PP‐1 and PP‐2A were established under screening by puromycin (1 μg/ml) for 4–6 weeks. The stable clones were first verified by Western blot. Note that knockdown of PP‐1β (a) and PP‐2Acα (b) but not PP‐1α (c), PP1γ (d), or PP‐2Acβ (e) leads to hyperphosphorylation of CREB at S133. (f & g) Overexpression of PP‐1β (f) or PP‐2Aα (g) through Tet‐on system induces hypophosphorylation of CREB at S133. (f & g) The stable PP‐1β (f) or PP‐2Acα (g) inducible αTN4‐1 cells grown to 80% confluence were pre‐incubated with 1, 50, 500, and 5000 ng/ml doxycyclin for 2 h. At the end, cells were harvested for analysis of PP‐1β (f) or PP‐2Acα (g) expression and phosphorylation status of CREB at S133 through Western blot analysis

To determine which isoforms of PP‐1 and PP‐2A phosphatases are implicated in dephosphorylation of CREB, we knocked down different isoforms of the catalytic subunits for PP‐1 and PP‐2A using plasmids generating shRNA for PP‐1α, PP‐1β, PP‐1γ, PP‐2Acα, and PP‐2Acβ. As shown in Figure 2a,b, when PP‐1β and PP‐2Acα were silenced with stable knockdown plasmids, both isoforms of phosphatases were clearly downregulated in their expression levels. Down‐regulation of PP‐1β and PP‐2Acα led to significant increase in the phosphorylation level of CREB at S133 while total CREB remained unchanged. Silence of 3 other isoforms caused little changes in the phosphorylation status of S133‐CREB (Figure 2c–e).

### 2.3 Overexpression of PP‐1β and PP‐2Aα through the Tet‐on system induces hypophosphorylation of CREB‐S133 in MLECs

To confirm that PP‐1β and PP‐2Aα are indeed the specific isoforms of phosphatases dephosphorylating CREB at S133, we overexpressed the two isoforms of the catalytic subunits of PP‐1 and PP‐2A using our previously established Tet‐on system (Li et al., 2006). As shown in Figure 2f,g, when doxycycline was increased to 500 ng/ml or higher, PP‐1β and PP‐2Acα were induced to a level that caused complete dephosphorylation of CREB at S133. These results clearly showed that both PP‐1β and PP‐2Aα are the major phosphatases that dephosphorylate CREB at S133. Thus, our data indicate that both PP‐1β and PP‐2Acα are capable to dephosphorylate CREB at S133.

### 2.4 MLECs expressing exogenous CREB and CREB‐S133A display differential ability to suppress expression of αB‐crystallin and contrast sensitivity to oxidative stress‐induced apoptosis

**FIGURE 3:**
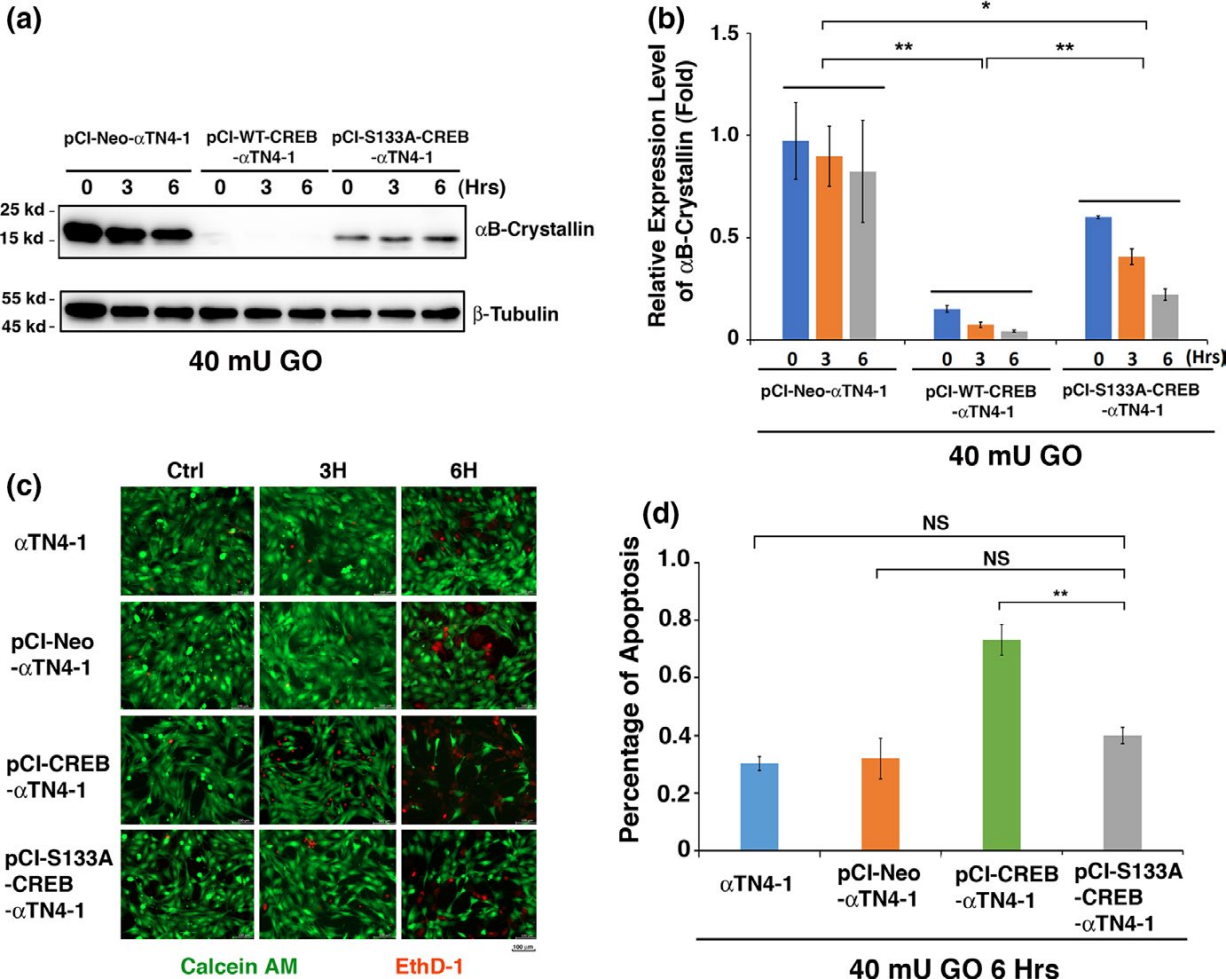
Expression of S133A mutant CREB significantly attenuates CREB Functions in suppressing αB‐crystallin expression and promoting hydrogen peroxide‐induced apoptosis. (a) Western blot analysis determined the expression levels of αB‐crystallin in pCI‐Neo‐αTN4‐1, pCI‐CREB‐αTN4‐1, and pCI‐S133A‐CREB‐αTN4‐1 cells under 40mU glucose oxidase (GO) treatment. (b) Semi‐quantification of the Western blot results in (a). (c) Live/dead assays analyze cell apoptosis induced by 40 mU GO, Green fluorescence represents live cells as detected by Calcein‐AM, and red fluorescence detected by EthD‐1 refers to dead cells. (d) CellTiter‐Glo^®^ luminescent cell viability assays to determine the rate of apoptosis. Note that pCI‐CREB‐αTN4‐1 cells displayed the highest level of apoptosis (about 80%) under 40 mU GO treatment. In contrast, cells expressing S133A‐CREB displayed much weak stress response to with less than half of apoptosis. All experiments were repeated three times. Error bar represents standard deviation, *N* = 3. NS, not significant, *p < 0.05, **p < 0.01

To compare whether CREB and S133A‐CREB have functional difference, we first established stable lines of MLECs expressing the empty vector, pCI‐αTN4‐1, wild‐type CREB, pCI‐CREB‐αTN4‐1, and also mutant CREB mimicking constant dephosphorylation at S133 residue, pCI‐S133A‐CREB‐αTN4‐1 (Figure S1). As we demonstrated recently (Wang et al., 2020), expression of exogenous wild‐type CREB completely suppressed expression of the αB‐Crystallin gene (Figure 3a). In contrast, expression of the exogenous mutant S133A‐CREB displayed significantly attenuated suppression of the αB‐Crystallin gene (Figure 3a,b).

Next, we treated four stable lines of MLECs, αTN4‐1, pCI‐αTN4‐1, pCI‐CREB‐αTN4‐1, and pCI‐S133A‐CREB‐αTN4‐1 with 40 mU glucose oxidase (GO) for 3–6 h (Figure 3c). During this process, GO induces production of hydrogen peroxide and reduction in protein thiols (Wang et al., 2020). Live and dead assay and ATP loss analysis (Crouch et al., 1993; Wang et al., 2020) revealed that cells expressing wild‐type CREB were most sensitive to GO‐induced apoptosis, and about 70% of the treated cells were undergoing apoptosis (Figure 3c,d). In contrast, in mutant CREB‐S133A expressing cells, less than 40% cells were found apoptotic, similar to those cells in parent and vector‐transfected cells (Figure 3c,d). Thus, our results revealed that S133A‐CREB has much attenuated functions than CREB in sensitizing lens epithelial cells to oxidative stress‐induced apoptosis.

### 2.5 RNAseq analysis revealed that expression of exogenous CREB altered the transcription patterns of some p53 target genes in MLECs

**FIGURE 4:**
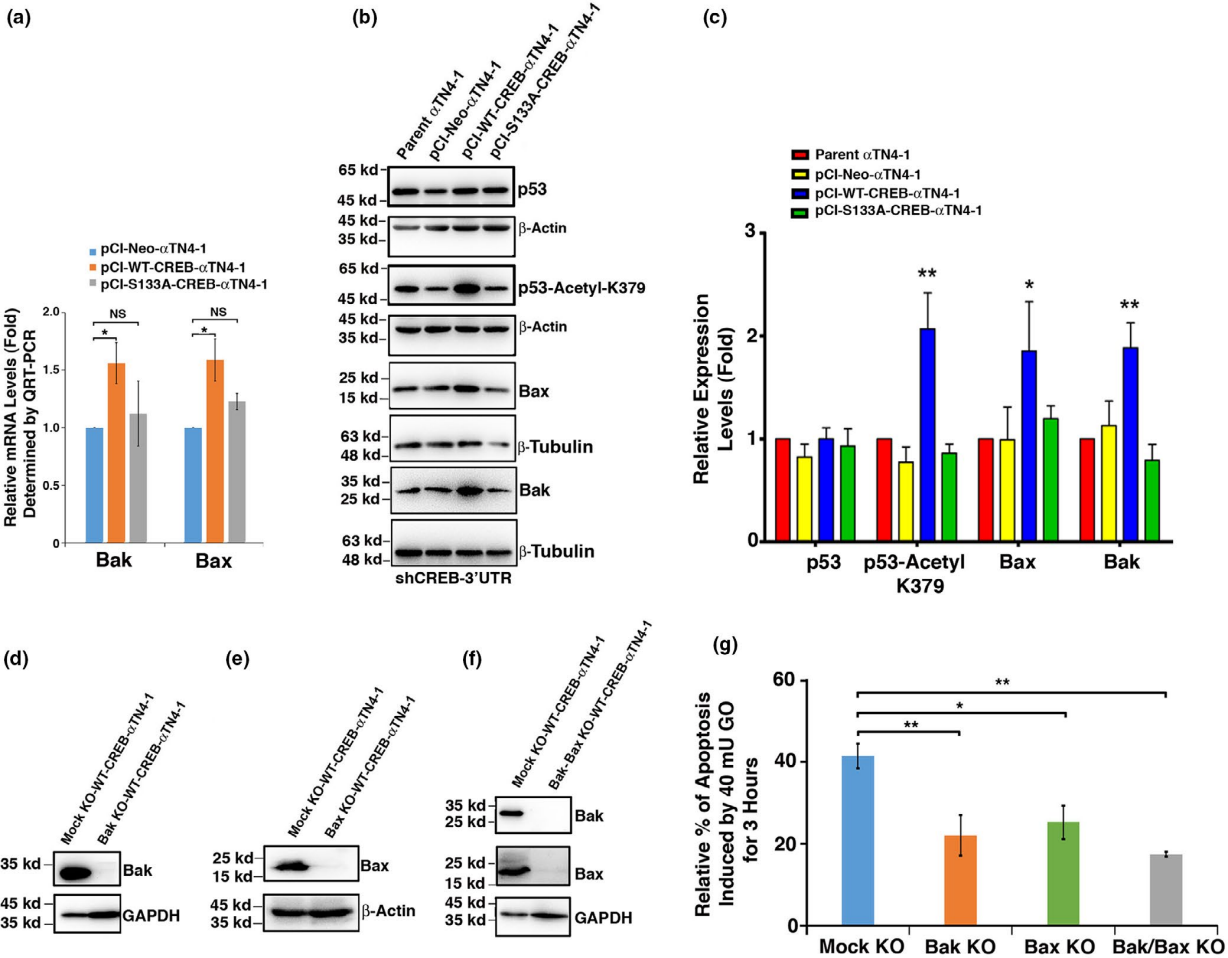
cAMP response element‐binding protein positively regulates pro‐apoptotic genes, Bax and Bak to enhance stress‐induced apoptosis. (a) QRT‐PCR confirms the RNAseq analysis data (Figure S2) to show both Bak and Bax are upregulated in cells expressing CREB but not the S133A‐CREB mutant. (b & c) Western blot analysis of the expression levels of P53, Acetyl‐p53‐K379, Bax, Bak, in parent αTN4‐1, pCI‐Neo‐αTN4‐1, pCI‐CREB‐αTN4‐1, and pCI‐S133A‐CREB‐αTN4‐1 cells, in all of which the endogenous CREB were knocked down with shRNA targeting to CREB 3′UTR (Figure S4). Note that the expression levels of Bak, Bax, and Acetyl‐p53‐K379 but not p53 were significantly upregulated in pCI‐CREB‐αTN4‐1 cell but not in pCI‐S133A‐CREB‐αTN4‐1 cells. (d, e, f, & g) CRISPR‐Cas9‐mediated Bak, Bax knockout or their double knockout significantly attenuated CREB‐promoted and 40 mU GO‐induced apoptosis of the pCI‐CREB‐αTN4‐1 cells with Bak, Bax, or Bak/Bax KO, respectively. (d, e, & f) WB confirms that Bak, Bax, or both of them were successfully knocked out from pCI‐CREB‐αTN4‐1 cell. (g) CellTiter‐Glo^®^ luminescent cell viability assay analysis determined the apoptosis rate in 40 mU GO‐induced mock‐, Bak‐knockout, Bax‐knockout, or Bak/Bax double knockout pCI‐CREB‐αTN4‐1 cells. Note that single knockout of either Bak or Bax can attenuate GO‐induced apoptosis, double knockout of Bak or Bax caused a further reduction in the CREB‐promoted and GO‐induced apoptosis of pCI‐CREB‐αTN4‐1 cells

To further understand why CREB and S133A‐CREB expression cells displayed differential sensitivity to oxidative stress‐induced apoptosis, we conducted RNAseq analysis with WT‐CREB and mutant S133A‐CREB‐transfected cells. As shown in Figure S2A, expression in a total of 857 genes displayed changes with 430 genes upregulated and 427 downregulated (SRA accession: PRJNA566306). Among these genes, besides the obviously changed expression pattern of αB‐crystallin (Figure S2B, Cryab), we noticed that the apoptosis‐related genes, the p53 target genes, Bak and Bax were also significantly changed (Figure S2B). QRT‐PCR analysis confirmed that Bak and Bax were upregulated in cells expressing CREB but not significant in S133A‐CREB cells (Figure 4a). QRT‐PCR analysis of eight other apoptosis‐related genes revealed absence of significant changes at the mRNA levels (Figure S3). To better compare the effects of CREB and S133A‐CREB, we silenced the endogenous CREB of the above parent and stable cell lines using shRNA targeting the 3′‐UTR sequences (Figure S4). Western blot analysis with these shCREB‐3′UTR cells lines further confirmed that CREB but not S133A‐CREB upregulated expression of Bak and Bax (Figure 4b,c). Thus, our results revealed that expression of exogenous CREB alters the expression patterns of a panel of apoptosis‐related genes including Bak and Bax besides its suppression of αB‐crystallin to mediate its hypersensitivity to oxidative stress‐induced apoptosis. The mutant S133A‐CREB, however, significantly lost the CREB function in upregulating Bak and Bax.

### 2.6 Knockdown of Bak, Bax, or both attenuates CREB‐promoted and GO‐induced apoptosis

To determine whether CREB‐mediated upregulation of Bak and Bax contributes to the hypersensitivity of the CREB expression cells to oxidative stress response, we have conducted CRISPR/Cas9‐mediated knockout of either Bak, Bax, or both of them in mouse lens epithelial cells. Bak, Bax, and Bak/Bax KO were verified by sequencing (Figure S5 and Western blot analysis (Figure 4d–f). When these CREB expression, Bak/Bax KO cells were then treated with 40 mU GO for 3 h, as shown in Figure 4g, KO of Bak, Bax, or both of them caused significant resistance to CREB‐promoted and GO‐induced apoptosis. In comparison with vector‐transfected cells, KO of Bak, Bax, or both of them caused even more significant resistance to GO‐induced apoptosis (data not shown). Thus, CREB‐regulated increase in Bak and Bax contributes to the hypersensitivity of CREB expression cells to oxidative stress‐induced apoptosis.

### 2.7 Overexpression of CREB but not CREB‐S133A upregulates p300 level in MLECs

**FIGURE 5:**
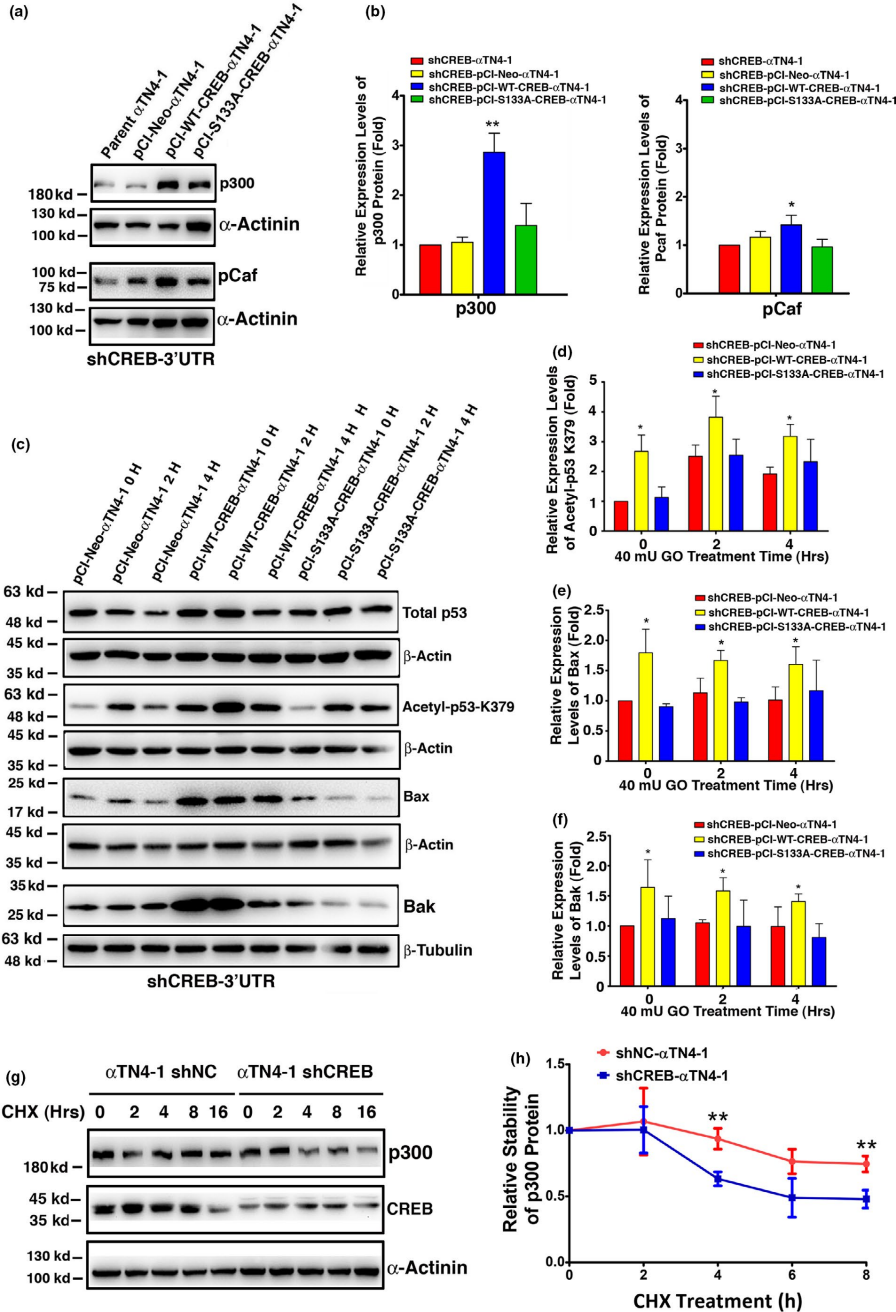
cAMP response element‐binding protein regulates p53 acetylation through control of p300 and Pcaf to promote upregulation of Bak and Bax. (a) Western blot analysis of two acetyltransferases, p300 and Pcaf, in parent αTN4‐1, pCI‐Neo‐αTN4‐1, pCI‐CREB‐αTN4‐1, and pCI‐S133A‐CREB‐αTN4‐1 cells with the endogenous CREB knocked down by shCREB‐3′UTR (Figure S4). (b) Semi‐quantification of the Western blot results in (a). Note that the expression of lysine acetyltransferase p300 was significantly upregulated in pCI‐CREB‐αTN4‐1 but not pCI‐S133A‐CREB‐αTN4‐1 cells. (c) Western blot analysis of total‐p53, acetyl‐p53‐k379, Bak, and Bax under 40 mU GO treatment in pCI‐Neo‐αTN4‐1, pCI‐CREB‐αTN4‐1, and pCI‐S133A‐CREB‐αTN4‐1 cells with the endogenous CREB knocked down by shCREB‐3′UTR (Figure S4). (d–f) Semi‐quantification of the Western blot results in (c). (g) Western blot analysis of p300 in cycloheximide (CHX)‐treated αTN4‐1‐shNC and αTN4‐1‐shCREB cells. (h) Semi‐quantification of the Western blot results in (g). Note that the half‐life of lysine acetyltransferase p300 was decreased in CREB knocked down αTN4‐1‐shCREB cells compared to mock knockdown αTN4‐1‐shNC cells. These results indicate that CREB regulates p300 stability to enhance p53 acetylation at K379

To understand why CREB causes upregulation of the pro‐apoptotic regulators, Bak and Bax, we first used bioinformatics to determine whether CREB can directly control p53 to upregulate Bak and Bax and found that p53 promoter does not contain CREB binding sites within 10 kb upstream and downstream (data not shown). Next, we checked if CREB could regulate p53 stability and activity through post‐translational modifications. Since the co‐activator, p300 acts as an acetyltransferase and has previously been shown to acetylate p53 (Gu & Roeder, 1997; Sakaguchi et al., 1998), we checked p300 levels and p53 acetylation status in pCI‐CREB‐αTN4‐1 and pCI‐S133A‐CREB‐αTN4‐1 cells with endogenous CREB knocked down by shRNA targeting 3′‐UTR sequences (Figure S4). As shown in Figure 5a,b, p300 was clearly upregulated in pCI‐CREB‐αTN4‐1 cells but not in pCI‐S133A‐CREB‐αTN4‐1 cells. We also examined the expression level of another p53 acetyltransferase, Pcaf, and found that it was upregulated in similar way as p300. Next, we checked the p53 acetylation status using Western blot analysis. As shown in Figures 4b,c and 5c,d, while total p53 remains unchanged in the two types of cells, p53 K379/K382 acetylation (in mouse K379 and in human K382) was significantly higher in pCI‐CREB‐αTN4‐1 cells than that in pCI‐S133A‐CREB‐αTN4‐1 cells without (Figure 4b,c) or with (Figure 5c,d) GO treatment. Consistent with the upregulation of p53 acetylation at K379/K382, the expression levels of both Bak and Bax were upregulated at each time point during GO treatment (Figure 5e,f). This is consistent with previous observation that phosphorylated CREB can recruit p300 to regulate CREB targets (Arany et al., 1994; Chrivia et al., 1993; Del et al., 2019; Ebrahimi et al., 2019). To understand how CREB upregulates p300, we have checked the promoter of p300 gene, and also its upstream and downstream sequences up to 10 kb regions, no conserved CREB binding site was identified (data not shown). This is consistent with our observation that CREB does not upregulate p300 mRNA (data not shown). Next, we examined if CREB increases p300 stability. To do so, cycloheximide was used to block protein synthesis in mock knockdown or CREB knockdown cells, and their p300 levels were analyzed by Western blot analysis. As shown in Figure 5g,h, CREB KD decreases the half‐life of p300. Thus, CREB interacts with p300 to increase its stability. Again, this is consistent with previous observation that phosphorylated CREB can recruit p300 to regulate CREB targets (Arany et al., 1994; Chrivia et al., 1993; Del et al., 2019; Ebrahimi et al., 2019).

### 2.8 CREB suppression of αB‐crystallin expression is observed in mouse model and in human cataractous lenses of different age groups

**FIGURE 6:**
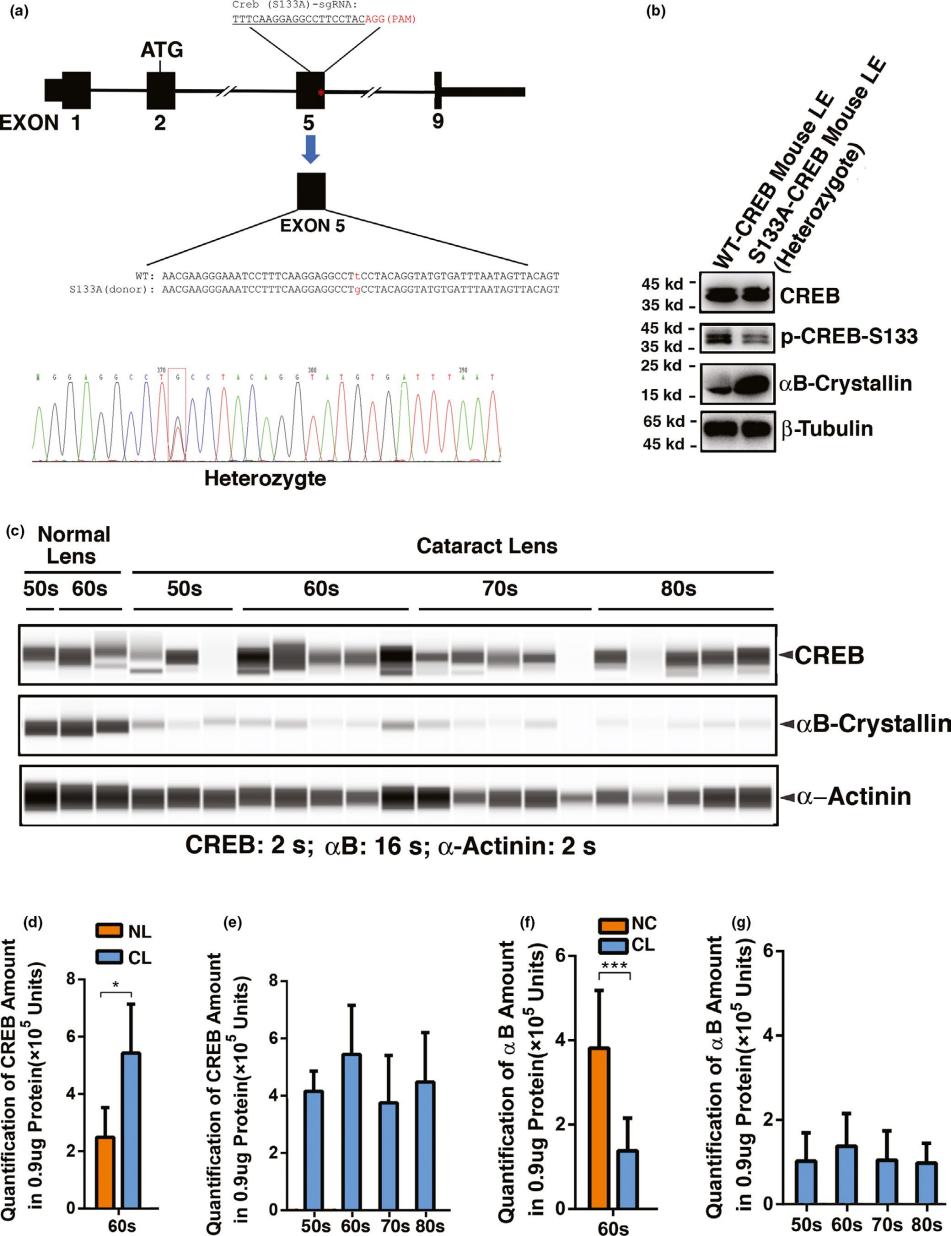
cAMP response element‐binding protein inhibits αB‐crystallin expression in mouse model as well as in cataract patients. (a) Schematic diagram of the strategy for generating the CREB‐S133A mutant heterozygote mouse strain by CRISPR/Cas9 genome editing. One set of sgRNA and donor DNA oligo were used to generate S133A mutant mice. The mapping sequence on mouse genomic DNA is shown. The S133A mice had a T changed to G (marked in red) in exon 5 (marked with red star symbol*). PAM, protospacer adjacent motif. (b) Western blot analysis of CREB, p‐CREB‐S133, and αB‐crystallin in lens epithelium of WT‐CREB and S133A‐CREB heterozygote mice. Note that as the CREB phosphorylation level became decreased in S133A mutant mice, the αB‐crystallin expression is greatly increased in these mice compared with the WT‐CREB mice. (c) The automated western immunoblot (AWI) analysis of CREB and αB‐crystallin in normal and cataractous lenses of different age groups. Automated Western immunoblot was performed on a Wes (ProteinSimple) as described recently (Dahl et al., 2016; Liu et al., 2020). Briefly, each sample was loaded with 0.9 μg total protein and then analyzed with the Size Separation Master Kit and Split Buffer (12–230 kDa) according to the manufacturer's standard instruction using anti‐CREB and anti‐αB‐crystallin antibody (for antibody information, see Experimental Procedures) with a dilution factor of 1:100 (anti‐CREB) or 1:1000 (anti‐αB‐crystallin). The Compass software (Protein Simple, version 4.1.5) was used to program the Wes and for presentation (c) and quantification (d–g). Output Western blot style data (c) were displayed with exposure time indicated, and the quantification data (d–g) were displayed from the software‐calculated average of seven exposures (1–512 s). (d & f) Quantification results show CREB (d) and αB‐crystallin (f) expression difference in human normal and cataract lenses of the 60s age group. Note compared to the normal lenses, the expression of CREB was significantly increased (d), in contrast, αB‐crystallin expression was greatly decreased in cataract lenses (f). NL, normal lenses; CL, cataractous lenses. (e & g) Quantification results show age difference of CREB and αB‐crystallin expression, respectively. Each bar represents an average of 12–18 cataract lens samples. *p < 0.05, ***p < 0.001

To confirm that WT‐CREB and S133A‐CREB indeed have differential functions in regulating αB‐crystallin gene, we generated a S133A‐CREB mouse model. Using CRISPR/Cas9 technology, a guide RNA targeting exon 5 converts a T to G conversion, resulting in the generation of the S133A‐CREB mice (Figure 6a). As shown in Figure 6b, S133A‐CREB heterozygous mice express a much higher level of αB‐crystallin than wild‐type mice do. Thus, CREB suppression of αB‐crystallin gene is confirmed in vivo.

To determine whether CREB suppression of αB‐crystallin expression is implicated in human cataractogenesis, we examined the relative levels of CREB and αB‐crystallin in human cataractous lenses of different age groups from 50s to 80s, and also compared with limited age‐matched normal human lenses. As shown in Figure 6c and Figure S6A, CREB level displayed age‐dependent decrease from 50s to 60s in normal human lenses. More importantly, significant CREB upregulation was observed from normal transparent lens to cataractous lenses of the 60s age group (Figure 6c,d). In cataract lenses from 50s to 80s, the CREB level becomes fluctuated with a level higher than that of the 60s transparent lenses (Figure 6e & Table S1). In contrast, αB‐crystallin level was significantly upregulated from 50s to 60s in normal human lenses but greatly downregulated from normal human lenses to cataractous lenses of the 60s age group (Figure 6c,f, Figure S6B & Table S1). In cataract lenses from 50s to 80s, αB‐crystallin level remains relatively stable with a level lower than that of the 60s transparent lenses (Figure 6g & Table S1). These results not only reveal that CREB suppression of αB‐crystallin as a pathological mechanism exists in human cataract lenses but also support our recent conclusion that CREB‐mediated repression of αB‐crystallin genes promotes stress‐induced apoptosis followed by cataractogenesis (Wang et al., 2020).

### 2.9 Acetylation of p53 at K382 and Pcaf expression are upregulated in human cataractous lenses of different age groups

**FIGURE 7:**
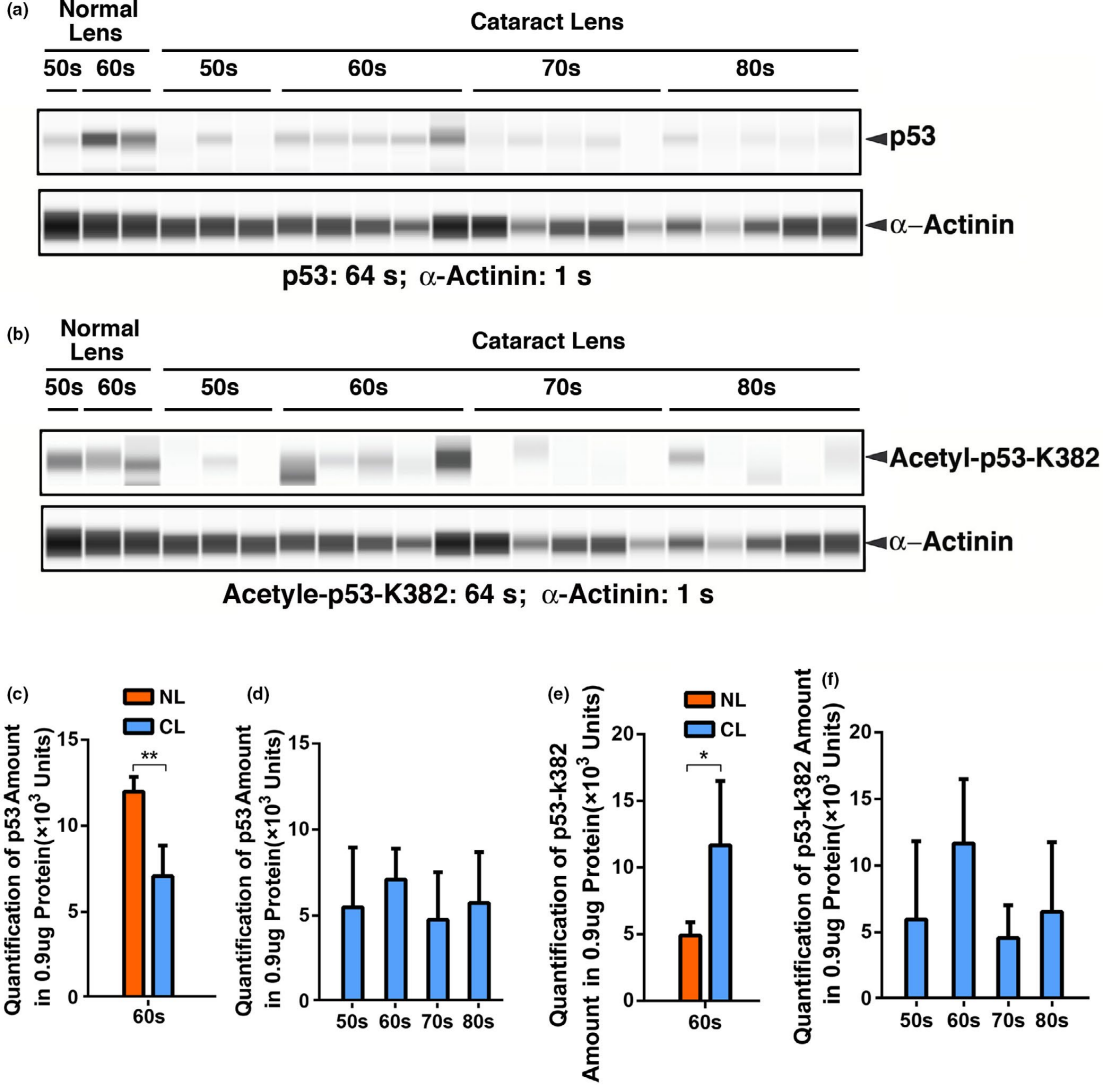
The automated Western immunoblot (AWI) analysis of p53 (a, c & d) and acetyl‐p53‐k382 (b, e & f) in normal and cataractous lenses of different age groups. (a) Output Western blot style data of p53 with exposure time indicated. (b) Output Western blot style data of acetyl‐p53‐k382 with exposure time indicated. (c–f) Quantification data derived from the software‐calculated average of seven exposures (1–512 s). (c & e) Quantification results show p53 (c) and p53‐K382 (e) expression difference in human normal and cataract lenses of the 60s age group. Note compared to the normal lenses, although the expression of p53 was significantly reduced (c), its acetylation at K382 was significantly enhanced in cataract lenses (e). NL, normal lenses; CL, cataractous lenses. (d & f) Quantification results show age difference of p53 and aceryl‐p53‐K382 expression. Each bar represents an average of 12–18 cataract lens samples. *p < 0.05, **p < 0.01

Since our work with mouse lens epithelial cells reveals that CREB can also promote upregulation of Bak and Bax through p300‐p53 signaling pathway, we next examined p53 acetylation in human cataractous samples of different age groups. As shown in Figure 7a,c, Figure S6C & Table S1, although p53 is downregulated from normal lens to cataractous lenses of the 60s age group, p53 acetylation at K379/K382 (the same antibody recognizes K379 in mouse and K382 in human) was significantly upregulated from normal lens to cataractous lenses of the same age group (Figure 7b,e, Figure S6D & Table S1). In cataractous lenses from 50s to 80s, p53 level was fluctuated slightly (Figure 7d & Table S1). The p53 acetylation at K379/K382 was upregulated from 50s to 60s and then returned to the level close to that of 50s from 60s to 80s (Figure 7f & Table S1). While p300 could not be checked under current experimental condition, we found that expression of Pcaf, another acetyltransferase for p53 was also significantly upregulated (Figure S7 & Table S1). Thus, CREB activation of p300‐p53 signal axis also plays some role in CREB regulation of stress response. In both wild‐type and S133A mice of 4 months, however, we failed to detect expression of p53 (data not shown).

### 2.10 Both PP‐1β and PP‐2Aα are downregulated in human cataractous lenses of different age groups

**FIGURE 8:**
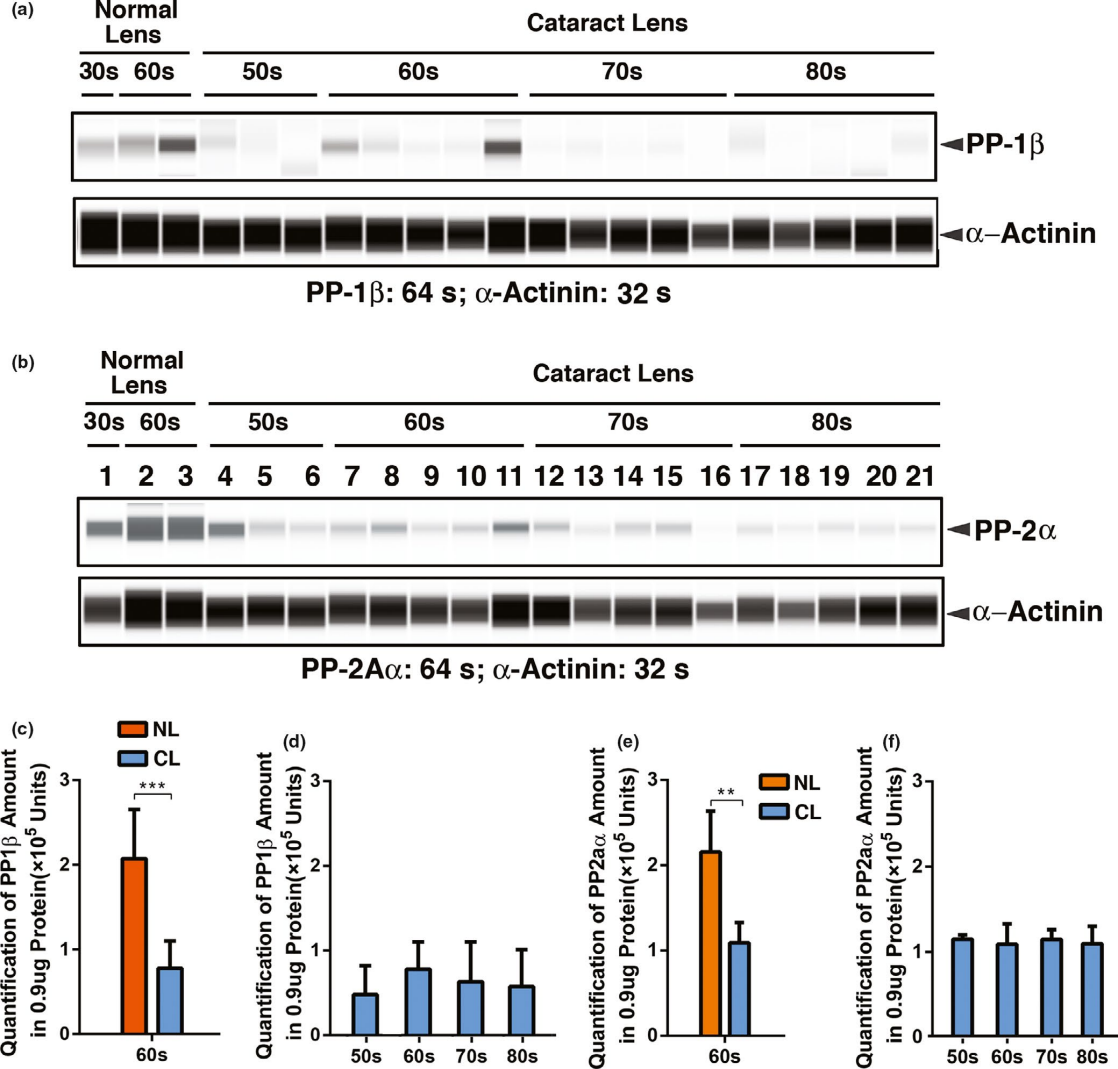
The automated Western immunoblot (AWI) analysis of PP1β (a, c & d) and PP2Aα (b, e & f) in normal and cataractous lenses of different age groups. (a & b) Output Western blot style data of PP1β and PP2Aα with exposure time indicated. (c–f) Quantification data derived from the software‐calculated average of seven exposures (1–512 s). (c & e) Quantification results show PP1β (c) and PP2Aα (e) expression difference in human normal and cataract lenses of the 60s age group. Note compared to the normal lenses, both the expression of PP1β (c) and PP2Aα (e) were significantly decreased. NL, normal lenses; CL, cataractous lenses. (d & f) Quantification results show age difference of PP1β and PP2Aα expression. Each bar represents an average of 12–18 cataract lens samples. **p < 0.01, ***p < 0.001

Since CREB is upregulated from normal transparent lenses to cataractous lenses of the 60s age group, we are curious about the changes of PP‐1β and PP‐2Aα. As shown in Figure 8 & Table S1, in normal human lenses, both PP‐1β and PP‐2Aα are upregulated from 30 to 60 years old. However, from transparent lenses to cataractous lenses in the 60s, these phosphatases are greatly downregulated. Down‐regulation of PP‐1β and PP‐2Aα could enhance CREB functions to suppress αB‐expression as observed in Figure 6c,d,f. Overexpression of the PP‐1β and PP‐2Aα in MLECs, on the contrary, suppresses CREB repression of αB‐crystallin expression (Figure S8). In cataractous lenses from 50s to 60s, PP‐1β level is upregulated and then becomes downregulated from 60s to 80s (Figure 8d). In contrast, PP‐2Aα level remains relatively stable from 50s to 80s (Figure 8f).

## 3 DISCUSSION

In the present study, we have demonstrated the followings: (1) Treatment of both lens epithelial cells, αTN4‐1 and mouse skin epithelial cells, JB6 with different concentrations of OA or LB100 causes dose‐dependent hyperphosphorylation of CREB at S133; (2) Silence of PP‐1β and PP‐2Aα causes hyperphosphorylation of CREB at S133; (3) Overexpression of PP‐1β and PP‐2Acα through the Tet‐on system leads to complete dephosphorylation of CREB. Together, our results for the first time defined the specific phosphatase isoforms that dephosphorylate CREB at S133. (4) CREB suppression of αB‐crystallin to promote stress‐induced apoptosis of lens epithelial cells can be largely inhibited in cells expressing the mutant CREB, S133A‐CREB; (5) S133A‐CREB mice express much higher level of αB‐crystallin in the ocular lens, confirming the in vivo suppression of αB‐crystallin by CREB; (6) RNAseq analysis, QRT‐PCR, and Western blot analysis demonstrated that CREB but not S133A‐CREB activates the p300‐p53 signaling axis and the downstream target genes, Bax and Bak; (7) Knockdown of Bax, Bak, or both of them increases resistance against oxidative stress‐induced apoptosis in CREB expression cells; (8) Both PP‐1β and PP‐2Aα are significantly upregulated in normal human lenses from 30 to 60 years old, which correlates with aging‐dependent down‐regulation of CREB. From normal transparent lenses to cataractous lenses of the 60s age group, however, both PP‐1β and PP‐2Aα are significantly downregulated. Their down‐regulation could enhance CREB suppression of αB‐crystallin and activation of p53 acetylation as observed (Figures 6, 7, 8‐8), implying that CREB‐regulated dual signaling pathways have important functions in aging control and lens pathology. It sensitizes lens epithelial cells to stress‐induced apoptosis by suppressing expression of the endogenous αB‐crystallin gene but promoting expression of Bax and Bak via p300‐p53 signaling pathways.

### 3.1 Both PP‐1 and PP‐2A can dephosphorylate CREB to modulate its functions

It has been well established that activation of CREB occurs through phosphorylation of S133 residue by the protein kinase A (PKA) (Altarejos & Montminy, 2011; Montminy et al., 1986). Inactivation of CREB is achieved by dephosphorylation through PP‐1 (Hagiwara et al., 1992) or PP‐2A (Wadzinski et al., 1993). A careful examination of the phosphatase‐mediated inactivation reveals presence of two unanswered questions: First, we noticed that inconsistent results have been reported in the literature regarding dephosphorylation through PP‐1 or PP‐2A. In PC12 cells, it was found that PP‐1 mediated CREB dephosphorylation at S133 caused down‐regulation of the downstream target, somatostatin gene (Hagiwara et al., 1992). This process can be inhibited by the specific PP‐1 inhibitor. On the contrary, in HepG2 cells, analyses with mono‐Q, amino‐hexyl sepharose, and heparin agarose columns revealed the association of CREB dephosphorylation with PP‐2A but in exclusion of PP‐1 (Wadzinski et al., 1993). Second, it was not known from these previous studies which isoforms of phosphatases are implicated in dephosphorylation of CREB. In the present study, using both lens and skin epithelial cells, we demonstrated that OA from low to high concentrations can cause dose‐dependent hyperphosphorylation of CREB (Figure 1), suggesting that both PP‐2A and PP‐1 are likely dephosphorylating CREB at S133. LB100 treatment further confirms the involvement of PP‐2A (Figure 1e,f). Subsequently, we conducted silence of different isoforms for both PP‐1 and PP‐2A using stable expression of the shRNAs from the transfected plasmids. We found that when PP‐1β or PP‐2Acα but not other isoforms of the catalytic subunits was knocked down, CREB phosphorylation at S133 was significantly enhanced (Figure 2a‐e). On the contrary, the Tet‐on induction of PP‐1β and PP‐2Acα expression led to complete dephosphorylation of CREB at S133 (Figure 2f,g). These results clearly demonstrate that both PP‐1 and PP‐2A can act on the CREB dephosphorylation at S133. Moreover, our results have determined that PP‐1β and PP‐2Acα are the two major isoforms of serine/threonine phosphatases that mediate dephosphorylation of CREB at this residue. Whether PP‐1β and PP‐2Acα also dephosphorylate CREB in other tissues remains to be further studied. Furthermore, our results demonstrate that dephosphorylation of CREB suppresses its function in mediating stress response and aging control (Figures 3, 4, 5, 6, see more discussion below).

### 3.2 Protein phosphorylation/dephosphorylation modulates functions of CREB and other key regulators to exert control on aging

Protein phosphorylation/dephosphorylation is a fundamental regulatory mechanism and controls the functions of more than one‐third of total eukaryotic proteins (Cohen, 1989; Hunter, 1995; Hunter & Karin, 1992; Moorhead et al., 2007; Mumby & Walter, 1993; Olsen et al., 2006). Through modulating major signaling transducers and transcription factors, protein phosphorylation/dephosphorylation plays important roles in regulating aging (Hart, 2019; Kang et al., 2013; Matsuoka et al., 2007; Pan & Finkel, 2017). In this regard, the tumor suppressor, p53, is an excellent example.

It is well established that DNA damage can cause genome instability, leading to acceleration of aging (Sperka et al., 2012). During DNA damaging, the initiating sensor is the ATM kinase encoded by the *ataxia* *telangiectasia*, which controls neuronal degeneration, hypersensitivity to ionizing radiation (IR), and premature aging (Zhou & Elledge, 2000). Activation of ATM through intermolecular autophosphorylation relays the signals to p53 by phosphorylating its Ser‐15 residue (Bakkenist & Kastan, 2003). The function of p53 is largely regulated by protein phosphorylation and dephosphorylation. At present, 17 serine/threonine residues of p53 have been identified and the activity of p53 is regulated by different kinases and phosphatases (Bode & Dong, 2004; Kruiswijk et al., 2015). We have previously demonstrated that both PP‐1 and PP‐2A can modulate p53 phosphorylation status at Ser‐15 and Ser‐37 to suppress its transcriptional activity and pro‐apoptotic activity (Li et al., 2006; Qin et al., 2008). Both p53 and the retinoblastoma protein (RB, another tumor suppressor) mediate two major pathways of aging (Campisi, 2005; Miura et al., 2004; Pelicci, 2004; Sperka et al., 2012; Stewart & Weinberg, 2006).

In the present study, we present the first evidence that CREB is significantly upregulated in cataractous lenses of more than 60 patients examined. The upregulated expression of CREB in the cataractous lenses suggests its functions in aging. Indeed, associated with CREB upregulation, we observed the significant down‐regulation of αB‐crystallin in these patients. Such results indicate CREB suppression of αB‐crystallin in human cataractous lenses, which is consistent with what we have recently revealed in rat lenses under stress insult (Wang et al., 2020). The CREB suppression of αB‐crystallin is further confirmed in the S133A‐CREB mice (Figure 6a) where the point mutation from Ser‐133 to Ala‐133 introduced by CRISPR/Cas9 technology in one copy of the gene leads to great upregulation of αB‐crystallin expression (Figure 6b). It has been shown that mice lacking αB‐crystallin display premature aging phenotypes. Brady et al. (2001) showed that at about 40 weeks of age, mice null for αB‐crystallin developed skeletal muscle atrophy, severe curvature of the spine, a significant loss of body fat and death at an earlier age compared to age‐matched wild‐type (WT) mice. These αB (−/−) mice appeared relatively normal compared to WT animals at younger ages, but at about 20 weeks of age muscle, fat, and bone deficiencies started to appear. These deficits are common in much older WT mice (Navarro et al., 2018). More recently, Lim et al. (2017) further show that αB‐crystallin expression correlates with aging deficits in peripheral nervous system. Thus, by suppressing αB‐crystallin expression in animal and human lenses, CREB acts as an important transcription factor promoting lens aging. Our results also show that the S133A‐CREB mutant mimicking constant CREB dephosphorylation at S133, on the contrary, had much attenuated ability in suppressing αB‐crystallin expression (Figures 3a and 6b), implying that both PP‐1 and PP‐2A likely slow down lens aging by de‐regulating CREB suppression of αB‐crystallin expression. Indeed, overexpression of PP‐1β or PP‐2Aα in MLECs promotes CREB S133 dephosphorylation and also upregulates expression of αB‐crystallin (Figure S8). Taken together, dephosphorylation regulation of CREB functions by PP‐1 and PP‐2A is implicated in control of aging.

### 3.3 Dephosphorylation of CREB by PP‐1β and PP‐2Aα promotes survival of lens epithelial cells

In the nerve system, CREB has been shown to mediate promotion of neuronal survival by NGF and BDGF (Bonni et al., 1999; Merk et al., 2018; Riccio et al., 1999). Mechanistically, the activated CREB can promote Bcl‐2 expression and thus enhance survival of the related neurons through activation of RSK90 kinase. In contrast, CREB plays a major role in stress response in the ocular lenses. By down‐regulating expression of αB‐crystallin, CREB promotes stress‐induced apoptosis (Wang et al., 2020). Besides its negative control of αB‐crystallin, in the present study, we found that CREB promotes stress‐induced apoptosis through activation of the p300‐p53 signaling axis. First, from RNAseq analysis followed by Western blot analysis and gene knockout, we demonstrated that the pro‐apoptotic genes, Bak and Bax, were upregulated in mouse lens epithelial cells expressing exogenous CREB (Figure 4). In deducing how CREB regulates Bak and Bax expression, we further demonstrated that CREB positively regulates p300 level through its interaction with the later to enhance its stability (Figure 5g,h). P300, being an acetyltransferase, has been shown to interact with p53 and promote its acetylation (Gu & Roeder, 1997; Sakaguchi et al., 1998). Indeed, we detected that CREB positively regulates p53 acetylation at K379 (Figure 5). In addition to p300, we also found that CREB upregulates expression of another acetyltransferase, Pcaf. How CREB regulates Pcaf is currently under investigation. Thus, through regulating p300 and other acetyltransferases, CREB promotes p53 acetylation to increase its transcription ability and upregulates expression of the pro‐apoptotic genes including Bax and Bak (Figures 4 and 5).

Our results further show that mouse lens epithelial cells expressing S133A‐CREB, on the contrary, display much lower level of apoptosis than those expressing CREB (Figure 3c,d). In analyzing the underlying mechanisms, we found that S133A‐CREB has much attenuated ability to suppress expression of αB‐crystallin both in vitro (Figure 3a,b) and in vivo (Figure 6b). In addition, S133A‐CREB no longer interacts with p300 to promote the stability of the later, and displays much weak ability to promote Pcaf expression (Figure 5a,b). As a result, the p300‐p53‐Bak/Bax signaling axis is not activated. More importantly, we found that p53‐K379/K382 acetylation and the acetyltransferase, Pcaf, are upregulated in human cataractous lenses (Figure 7 and Figure S7), confirming the importance of the CREB activation of the p300‐p53 signaling axis in human lens pathology. Taken together, dephosphorylation of CREB by PP‐1β and PP‐2Acα de‐regulates its suppression of αB‐crystallin expression and activation of p300‐p53‐Bak/Bax signaling axis to promote survival of lens epithelial cells.

### 3.4 CREB, αB‐crystallin, PP‐1β, and PP‐2Aα may be used for molecular signature of human senile cataracts

The ocular lens is an excellent organ to study aging because of its simplicity in structure containing only two types of cells, the anterior single layer of epithelial cells and the differentiating or differentiated fiber cells (Li et al., 2005). Lack of any distribution of vascular and nerve tissue in the mature lenses makes it even simpler. In addition, our previous study demonstrated that the lens epithelial cells have the similarly conserved signaling pathways but distinct functions (Li et al., 2005). For example, the stress‐activated Ras/Raf/MEK/ERK pathway confers survival in non‐lens cells (Xia et al., 1995) but mediates stress‐induced apoptosis in lens epithelial cells (Li et al., 2005).

During aging of the ocular lens, extensive changes have been detected in major lens structure proteins, members of different families of crystallins including α‐, β‐, and γ‐crystallins (Friedrich et al., 2017; Quinlan & Hogg, 2018; Su et al., 2012; Truscott & Friedrich, 2016; Wang et al., 2014, 2019). In this regard, Su et al. (2012) observed that autolytic cleavage of crystallins adjacent to serine residues yields about 25% of all peptides derived from αA‐, αB‐, β‐, and γS‐crystallins, which may act as the molecular signatures of long‐lived proteins in the ocular lens. In a subsequent study, Wang et al. (2014) suggest that non‐enzymatic post‐translational modifications (PTM) of the lens structure proteins seem to be a fundamental molecular process of aging since the combination of various modifications and their accumulation with age not only affects function, but leads to crosslinking and protein aggregation, thus causing light scattering.

Besides structure proteins, molecular changes found in various enzymes responsible for cellular redox setting, cytoskeletons, and intermediate filaments induced by oxidative and other stress conditions are also linked to aging and lens pathology (Barnes & Quinlan, 2017; Fan et al., 2017; Giblin et al., 1995; Raghavan et al., 2016; Rakete & Nagaraj, 2016; Reddy et al., 2001; Wang et al., 2017). More recently, Wang et al. (2017) identified 74 and 50 disulfide‐forming proteins in human and mouse cataractous lenses among which a majority of proteins are redoxing enzymes. In addition, the same group reported that extensive oxidation was detected in lens‐specific intermediate filament proteins (Wang et al., 2017).

More recently, we reported that the sumoylation enzyme systems display distinct changes during aging and cataractogenesis (Liu et al., 2020). The ligases, UBA2, Ubc9, and PIAS1, as well as the de‐sumoylation enzyme SENP2/6 are found upregulated in human cataractous lenses from 50s to 70s of different age groups. All sumoylation enzymes, however, are downregulated from 70s to 80s. Thus, these enzymes can be used as molecular markers for senile cataract. Among sumoylation substrates, we have also demonstrated that the contrast stability of p46 and p32 Pax6 can be used as another molecular marker for senile cataract. In the present study, we have found that in cataractous lenses from 66 patients of different age groups, expression of CREB, αB‐crystallin, PP‐1β, and PP‐2Aα display unique patterns and thus can also be used for molecular markers for senile cataract.

**FIGURE 9:**
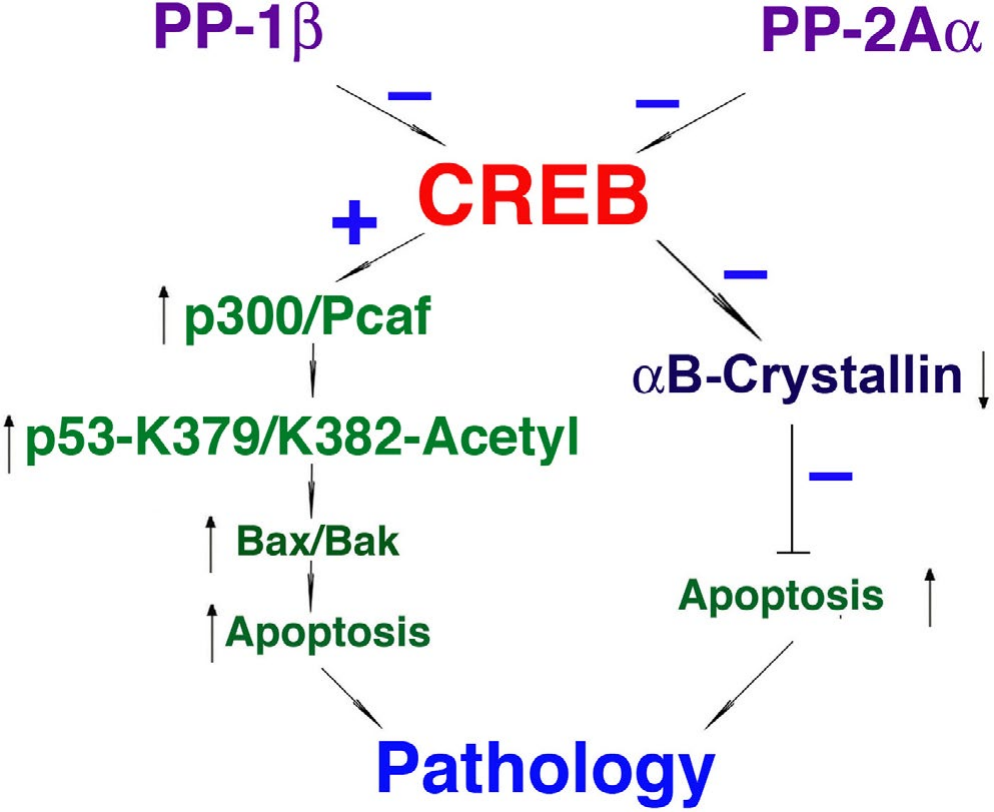
A schematic diagram to show that CREB can promote aging and stress‐induced apoptosis through two major signaling mechanisms: direct suppression of αB‐crystallin, and indirect activation of p53 acetylation and pro‐apoptotic genes, Bax/Bak. Suppression of αB‐crystallin, an anti‐aging and anti‐apoptotic regulator causes apoptosis of lens epithelial cells; on the contrary, p53 acetylation promotes upregulation of pro‐apoptotic genes, both Bax and Bak to promote stress‐induced apoptosis. As a result, both pathways merged to cause ocular pathogenesis–cataractogenesis. These pathways are clearly detected in human cataractous lenses (Figures 6, 7, 8). cAMP response element‐binding protein functions in both aspects are negatively modulated by PP‐1β and PP‐2Aα through dephosphorylation at S133

In summary, the results presented in this study reveal important functions of CREB in mediating aging control and stress response. Through suppression of αB‐crystallin expression and activation of the p300‐p53‐Bak/Bax signaling axis, CREB promote aging and apoptosis. Protein phosphatases, both PP‐1β and PP‐2Aα, can modulate CREB functions by dephosphorylating S133 residue. Dephosphorylation of CREB at S133 de‐regulates CREB suppression of αB‐crystallin expression and, to a less degree of importance, modulates the p300‐p53 signaling axis. In this way, PP‐1 and PP‐2A promote survival of lens epithelial cells and also slow down aging process (Figure 9).

## 4 EXPERIMENTAL PROCEDURES

### 4.1 Chemicals

Detailed information about chemicals used in this study is described in the Appendix S1.

### 4.2 Collection of human lens capsular epithelial samples

Collection of human capsular epithelia from cataract lenses of different age groups was approved by the Institutional Review Board of the Zhongshan Ophthalmic Center (ZOC). Informed written consent was obtained from each of the cataract patients. Detailed information is described in the Appendix S1.

### 4.3 CRISPR/Cas9‐mediated gene editing to generate S133A‐CREB mouse model

The S133A‐CREB heterozygote mouse model was generated using CRISPR/Cas9 technology as described before (Gong et al., 2018). Detailed information is described in the Appendix S1.

### 4.4 Culture of mouse lens epithelial cells (αTN4‐1), and mouse skin epithelial cells (JB6) and their treatment by okadaic acid (OA) and LB100

Culture of mouse lens epithelial cells (αTN4‐1), and mouse skin epithelial cells (JB6) and their treatment by okadaic acid (OA) and LB100 were conducted as we described before (Li et al., 2006; Qin et al., 2008). Detailed information is described in the Appendix S1.

### 4.5 Silence of PP‐1 or PP‐2A subunits and overexpression of PP‐1β and PP‐2Acα in αTN4‐1 cells

Silence of PP‐1 or PP‐2A subunits and overexpression of PP‐1β and PP‐2Acα through Tet‐on induction system or using pCI‐Neo Vector‐carried cDNA in αTN4‐1 cells were conducted as we described before (Li et al., 2006; Xiao et al., 2010). Detailed information is described in the Appendix S1.

### 4.6 Establishment of stable expression cell lines

The establishment of stable expression cell lines, pCI‐Neo‐αTN4‐1, pCI‐CREB‐αTN4‐1, and pCI‐S133A‐CREB‐αTN4‐1, was conducted as we described before (Wang et al., 2020). Detailed information is described in the Appendix S1.

### 4.7 RNA interference and lentivirus infection

The shRNA‐mediated CREB knockdown was conducted using the pLKO lentiviral expression system targeting the 3′UTR region of CREB gene. Detailed information is described in the Appendix S1.

### 4.8 Treatment by 40 mU Glucose oxidase (GO)

Various lines of cells were treated by 40 mU Glucose oxidase (GO) as we described (Gong et al., 2018; Wang et al., 2020). Detailed information is described in the Appendix S1.

### 4.9 Apoptosis analysis with cellTiter‐Glo^®^ luminescent cell viability assay and live/dead viability/cytotoxicity

The percentage of apoptosis in GO‐treated mouse lenses were determined by cellTiter‐Glo^®^ luminescent cell viability assay kit (Promega, G7573) as described before (Crouch et al., 1993). Detailed information is described in the Appendix S1.

### 4.10 Total protein extraction and Western blot analysis

Total proteins were extracted from cultured lens epithelial cells and capsular epithelial samples using RIPA buffer, processed for Western blot analysis as we described before (Gong et al., 2014; Gong et al., 2018; Li et al., 1995, 2005, 2006; Li & Spector, 1996; Mao et al., 2004; Qin et al., 2008; Xiao et al., 2010). Detailed information is described in the Appendix S1.

### 4.11 Automated western immunoblotting

The simple western immunoblots were performed on a Wes (ProteinSimple) as previously described (Dahl et al., 2016; Liu et al., 2020). Detailed information is described in the Appendix S1.

### 4.12 RT‐PCR, qRT‐PCR, and RNAseq Analysis

RT‐PCR, qRT‐PCR, and RNAseq analysis were conducted as we described before (Wang et al., 2020). Detailed information is described in the Appendix S1.

### 4.13 Establishment of Bak, Bax, and Bak/Bax knockout stable cell lines

Establishment of Bak, Bax, and Bak/Bax knockout stable cell lines was conducted using CRISPR/Cas9 system. Detailed information is described in the Appendix S1.

### 4.14 DNA sequencing

Verification of the Bak, Bax, and Bak/Bax knockout stable cell lines was conducted using DNA sequencing. Detailed information is described in the Appendix S1.

### 4.15 Statistical analysis

All experiments were repeated at least three times (*N* = 3) except for RNAseq analysis in which each analyzed sample was a pool of two separated samples (*N* = 4). Significance was determined by two‐tailed Student's *t*‐test. The error bar in all figures represents standard deviation. The *p* value < 0.05 was considered statistically significant. *, **, and *** represent *p* < 0.05, 0.01, and 0.001, respectively.

## CONFLICT OF INTEREST

None declared.

## AUTHOR CONTRIBUTIONS

LW, YZL, and DW‐CL designed research. LW, XDG, LZ, JLF, YWG, JWX, YX, YW, SYZ, and LY performed the experiments. QN, MH, and HMC coordinated collection of capsular epithelia samples from surgeons. LW, LZ, XDG, JLF, YWG and DW‐CL analyzed the data. LW and DW‐CL wrote the paper.

## PATIENT CONSENT

Patient consent has been obtained for all patients.

## Supporting information

Supplementary Material

## Data Availability

All data are available upon request. Raw data and processed data will be made available at Gene Expression Omnibus (https://www.ncbi.nlm.nih.gov) upon acceptance of the manuscript for publication.

## References

[acel13458-bib-0001] Altarejos, J. Y., & Montminy, M. (2011). CREB and the CRTC co‐activators: Sensors for hormonal and metabolic signals. Nature Reviews Molecular Cell Biology, 12, 141–151. 21346730 10.1038/nrm3072 PMC4324555

[acel13458-bib-0002] Andley, U. P., Song, Z., Wawrousek, E. F., Fleming, T. P., & Bassnett, S. (2000). Differential protective activity of alpha A‐ and alphaB‐crystallin in lens epithelial cells. Journal of Biological Chemistry, 275, 36823–36831. 10.1074/jbc.M004233200 10967101

[acel13458-bib-0003] Arany, Z., Sellers, W. R., Livingston, D. M., & Eckner, R. (1994). E1A‐associated p300 and CREB‐associated CBP belong to a conserved family of coactivators. Cell, 77(6), 799–800. 10.1016/0092-8674(94)90127-9 8004670

[acel13458-bib-0004] Bakkenist, C. J., & Kastan, M. B. (2003). DNA damage activates ATM through intermolecular autophosphorylation and dimer dissociation. Nature, 421, 499–506. 12556884 10.1038/nature01368

[acel13458-bib-0005] Barnes, S., & Quinlan, R. A. (2017). Small molecules, both dietary and endogenous, influence the onset of lens cataracts. Experimental Eye Research, 156, 87–94. 27039707 10.1016/j.exer.2016.03.024 PMC5107344

[acel13458-bib-0006] Bartsch, D., Casadio, A., Karl, K. A., Serodio, P., & Kandel, E. R. (1998). CREB1 encodes a nuclear activator, a repressor, and a cytoplasmic modulator that form a regulatory unit critical for long‐term facilitation. Cell, 95(2), 211–223. 10.1016/S0092-8674(00)81752-3 9790528

[acel13458-bib-0007] Bloemendal, H. (1991). Proctor lecture. Disorganization of membranes and abnormal intermediate filament assembly lead to cataract. Investigative Ophthalmology & Visual Science, 32, 445–455. 2001920

[acel13458-bib-0008] Bode, A. M., & Dong, Z. (2004). Post‐translational modification of p53 in tumorigenesis. Nature Reviews Cancer, 4, 793–805. 15510160 10.1038/nrc1455

[acel13458-bib-0009] Bonni, A., Brunet, A., West, A. E., Datta, S. R., Takasu, M. A., & Greenberg, M. E. (1999). Cell survival promoted by the Ras‐MAPK signaling pathway by transcription‐dependent and ‐independent mechanisms. Science, 286, 1358–1362. 10.1126/science.286.5443.1358 10558990

[acel13458-bib-0010] Bourtchuladze, R., Frenguelli, B., Blendy, J., Cioffi, D., Schutz, G., & Silva, A. J. (1994). Deficient long‐term memory in mice with a targeted mutation of the cAMP‐responsive element‐binding protein. Cell, 79, 59–68. 10.1016/0092-8674(94)90400-6 7923378

[acel13458-bib-0011] Brady, J. P., Garland, D. L., Green, D. E., Tamm, E. R., Giblin, F. J., & Wawrousek, E. F. (2001). AlphaB‐crystallin in lens development and muscle integrity: A gene knockout approach. Investigative Ophthalmology & Visual Science, 42, 2924–2934. 11687538

[acel13458-bib-0012] Campisi, J. (2005). Senescent cells, tumor suppression, and organismal aging: Good citizens, bad neighbors. Cell, 120, 513–522. 10.1016/j.cell.2005.02.003 15734683

[acel13458-bib-0013] Chrivia, J. C., Kwok, R. P., Lamb, N., Hagiwara, M., Montminy, M. R., & Goodman, R. H. (1993). Phosphorylated CREB binds specifically to the nuclear protein CBP. Nature, 365, 855–859. 10.1038/365855a0 8413673

[acel13458-bib-0014] Cogan, D. G. (1973). The lens, cataracts, and galactosemia. New England Journal of Medicine, 288, 1239–1240. 10.1056/NEJM197306072882313 4700558

[acel13458-bib-0015] Cohen, P. (1989). The structure and regulation of protein phosphatases. Annual Review of Biochemistry, 58, 453–508. 10.1146/annurev.bi.58.070189.002321 2549856

[acel13458-bib-0016] Comb, M., Birnberg, N. C., Seasholtz, A., Herbert, E., & Goodman, H. M. (1986). A cyclic AMP‐ and phorbol ester‐inducible DNA element. Nature, 323, 353–356. 10.1038/323353a0 3020428

[acel13458-bib-0017] Crouch, S. P., Kozlowski, R., Slater, K. J., & Fletcher, J. (1993). The use of ATP bioluminescence as a measure of cell proliferation and cytotoxicity. Journal of Immunological Methods, 160, 81–88. 10.1016/0022-1759(93)90011-U 7680699

[acel13458-bib-0018] Dahl, J. A., Jung, I., Aanes, H., Greggains, G. D., Manaf, A., Lerdrup, M., Li, G., Kuan, S., Li, B., Lee, A. Y., Preissl, S., Jermstad, I., Haugen, M. H., Suganthan, R., Bjoras, M., Hansen, K., Dalen, K. T., Fedorcsak, P., Ren, B., & Klungland, A. (2016). Broad histone H3K4me3 domains in mouse oocytes modulate maternal‐to‐zygotic transition. Nature, 537, 548–552. 27626377 10.1038/nature19360 PMC6283663

[acel13458-bib-0019] Del, B. B., Guiretti, D., Tomasoni, R., Lopez‐Cascales, M. T., Munoz‐Viana, R., Lipinski, M., Scandaglia, M., Coca, Y., Olivares, R., Valor, L. M., Herrera, E., & Barco, A. (2019). CBP and SRF co‐regulate dendritic growth and synaptic maturation. Cell Death and Differentiation, 26, 2208–2222. 30850733 10.1038/s41418-019-0285-x PMC6889142

[acel13458-bib-0020] Ebrahimi, A., Sevinc, K., Gurhan, S. G., Cribbs, A. P., Philpott, M., Uyulur, F., Morova, T., Dunford, J. E., Goklemez, S., Ari, S., Oppermann, U., & Onder, T. T. (2019). Bromodomain inhibition of the coactivators CBP/EP300 facilitate cellular reprogramming. Nature Chemical Biology, 15, 519–528. 10.1038/s41589-019-0264-z 30962627 PMC6504645

[acel13458-bib-0021] Fan, X., Monnier, V. M., & Whitson, J. (2017). Lens glutathione homeostasis: Discrepancies and gaps in knowledge standing in the way of novel therapeutic approaches. Experimental Eye Research, 156, 103–111. 10.1016/j.exer.2016.06.018 27373973 PMC5199622

[acel13458-bib-0022] Fernandez, J. J., Candenas, M. L., Souto, M. L., Trujillo, M. M., & Norte, M. (2002). Okadaic acid, useful tool for studying cellular processes. Current Medicinal Chemistry, 9, 229–262. 11860357 10.2174/0929867023371247

[acel13458-bib-0023] Friedrich, M. G., Wang, Z., Oakley, A. J., Schey, K. L., & Truscott, R. (2017). Hotspots of age‐related protein degradation: The importance of neighboring residues for the formation of non‐disulfide crosslinks derived from cysteine. The Biochemical Journal, 474, 2475–2487. 10.1042/BCJ20170268 28592682 PMC5685506

[acel13458-bib-0024] Gong, L., Ji, W. K., Hu, X. H., Hu, W. F., Tang, X. C., Huang, Z. X., Li, L., Liu, M., Xiang, S. H., Wu, E., Woodward, Z., Liu, Y. Z., Nguyen, Q. D., & Li, D. W. (2014). Sumoylation differentially regulates Sp1 to control cell differentiation. Proceedings of the National Academy of Sciences of the United States of America, 111, 5574–5579. 24706897 10.1073/pnas.1315034111 PMC3992630

[acel13458-bib-0025] Gong, L., Liu, F., Xiong, Z., Qi, R., Luo, Z., Gong, X., Nie, Q., Sun, Q., Liu, Y. F., Qing, W., Wang, L., Zhang, L., Tang, X., Huang, S., Li, G., Ouyang, H., Xiang, M., Nguyen, Q. D., Liu, Y., & Li, D. W. (2018). Heterochromatin protects retinal pigment epithelium cells from oxidative damage by silencing p53 target genes. Proceedings of the National Academy of Sciences of the United States of America, 115, E3987–E3995. 29622681 10.1073/pnas.1715237115 PMC5924883

[acel13458-bib-0026] Giblin, F. J., Padgaonkar, V. A., Leverenz, V. R., Lin, L. R., Lou, M. F., Unakar, N. J., Dang, L., Dickerson, J. J., & Reddy, V. N. (1995). Nuclear light scattering, disulfide formation and membrane damage in lenses of older guinea pigs treated with hyperbaric oxygen. Experimental Eye Research, 60, 219–235. 10.1016/S0014-4835(05)80105-8 7789403

[acel13458-bib-0027] Gu, W., & Roeder, R. G. (1997). Activation of p53 sequence‐specific DNA binding by acetylation of the p53 C‐terminal domain. Cell, 90, 595–606. 9288740 10.1016/s0092-8674(00)80521-8

[acel13458-bib-0028] Hagiwara, M., Alberts, A., Brindle, P., Meinkoth, J., Feramisco, J., Deng, T., Karin, M., Shenolikar, S., & Montminy, M. (1992). Transcriptional attenuation following cAMP induction requires PP‐1‐mediated dephosphorylation of CREB. Cell, 70, 105–113. 10.1016/0092-8674(92)90537-M 1352481

[acel13458-bib-0029] Hagiwara, M., Brindle, P., Harootunian, A., Armstrong, R., Rivier, J., Vale, W., Tsien, R., & Montminy, M. R. (1993). Coupling of hormonal stimulation and transcription via the cyclic AMP‐responsive factor CREB is rate limited by nuclear entry of protein kinase A. Molecular and Cellular Biology, 13, 4852–4859. 10.1128/MCB.13.8.4852 8336722 PMC360117

[acel13458-bib-0030] Hart, G. W. (2019). Nutrient regulation of signaling and transcription. Journal of Biological Chemistry, 294, 2211–2231. 10.1074/jbc.AW119.003226 PMC6378989 30626734

[acel13458-bib-0031] Hunter, T. (1995). Protein kinases and phosphatases: The yin and yang of protein phosphorylation and signaling. Cell, 80, 225–236. 10.1016/0092-8674(95)90405-0 7834742

[acel13458-bib-0032] Hunter, T., & Karin, M. (1992). The regulation of transcription by phosphorylation. Cell, 70, 375–387. 1643656 10.1016/0092-8674(92)90162-6

[acel13458-bib-0033] Kang, S. A., Pacold, M. E., Cervantes, C. L., Lim, D., Lou, H. J., Ottina, K., Gray, N. S., Turk, B. E., Yaffe, M. B., & Sabatini, D. M. (2013). mTORC1 phosphorylation sites encode their sensitivity to starvation and rapamycin. Science, 341, 1236566. 23888043 10.1126/science.1236566 PMC3771538

[acel13458-bib-0034] Kim, W. Y., & Sharpless, N. E. (2006). The regulation of INK4/ARF in cancer and aging. Cell, 127, 265–275. 17055429 10.1016/j.cell.2006.10.003

[acel13458-bib-0035] Kruiswijk, F., Labuschagne, C. F., & Vousden, K. H. (2015). p53 in survival, death and metabolic health: A lifeguard with a licence to kill. Nature Reviews Molecular Cell Biology, 16, 393–405. 10.1038/nrm4007 26122615

[acel13458-bib-0036] Kruse, J. P., & Gu, W. (2009). Modes of p53 regulation. Cell, 137, 609–622. 19450511 10.1016/j.cell.2009.04.050 PMC3737742

[acel13458-bib-0037] Li, D. W., Liu, J. P., Mao, Y. W., Xiang, H., Wang, J., Ma, W. Y., Dong, Z., Pike, H. M., Brown, R. E., & Reed, J. C. (2005). Calcium‐activated RAF/MEK/ERK signaling pathway mediates p53‐dependent apoptosis and is abrogated by alpha B‐crystallin through inhibition of RAS activation. Molecular Biology of the Cell, 16, 4437–4453. 16000378 10.1091/mbc.E05-01-0010 PMC1196350

[acel13458-bib-0038] Li, D. W., Liu, J. P., Schmid, P. C., Schlosser, R., Feng, H., Liu, W. B., Yan, Q., Gong, L., Sun, S. M., Deng, M., & Liu, Y. (2006). Protein serine/threonine phosphatase‐1 dephosphorylates p53 at Ser‐15 and Ser‐37 to modulate its transcriptional and apoptotic activities. Oncogene, 25, 3006–3022. 16501611 10.1038/sj.onc.1209334

[acel13458-bib-0039] Li, W. C., Kuszak, J. R., Dunn, K., Wang, R. R., Ma, W., Wang, G. M., Spector, A., Leib, M., Cotliar, A. M., Weiss, M., & Et, A. (1995). Lens epithelial cell apoptosis appears to be a common cellular basis for non‐congenital cataract development in humans and animals. Journal of Cell Biology, 130, 169–181. 10.1083/jcb.130.1.169 PMC2120521 7790371

[acel13458-bib-0040] Li, W. C., & Spector, A. (1996). Lens epithelial cell apoptosis is an early event in the development of UVB‐induced cataract. Free Radical Biology and Medicine, 20, 301–311. 8720900 10.1016/0891-5849(96)02050-3

[acel13458-bib-0041] Lim, E. F., Musa, A., Frederick, A., & Ousman, S. S. (2017). AlphaB‐crystallin expression correlates with aging deficits in the peripheral nervous system. Neurobiology of Aging, 53, 138–149. 10.1016/j.neurobiolaging.2017.01.006 28185662

[acel13458-bib-0042] Lim, J. C., Grey, A. C., Zahraei, A., & Donaldson, P. J. (2020). Age‐dependent changes in glutathione metabolism pathways in the lens: New insights into therapeutic strategies to prevent cataract formation‐A review. Clinical & Experimental Ophthalmology, 48, 1031–1042. 10.1111/ceo.13801 32462803

[acel13458-bib-0043] Liu, F. Y., Fu, J. L., Wang, L., Nie, Q., Luo, Z., Hou, M., Yang, Y., Gong, X. D., Wang, Y., Xiao, Y., Xiang, J., Hu, X., Zhang, L., Wu, M., Chen, W., Cheng, B., Luo, L., Zhang, X., Liu, X., … Li, D. W. (2020). Molecular signature for senile and complicated cataracts derived from analysis of sumoylation enzymes and their substrates in human cataract lenses. Aging Cell, 19, e13222. 10.1111/acel.13222 32827359 PMC7576240

[acel13458-bib-0044] Mao, Y. W., Liu, J. P., Xiang, H., & Li, D. W. (2004). Human alphaA‐ and alphaB‐crystallins bind to Bax and Bcl‐X(S) to sequester their translocation during staurosporine‐induced apoptosis. Cell Death and Differentiation, 11, 512–526. 14752512 10.1038/sj.cdd.4401384

[acel13458-bib-0045] Martin, J. L., Mestril, R., Hilal‐Dandan, R., Brunton, L. L., & Dillmann, W. H. (1997). Small heat shock proteins and protection against ischemic injury in cardiac myocytes. Circulation, 96, 4343–4348. 10.1161/01.CIR.96.12.4343 9416902

[acel13458-bib-0046] Matsuoka, S., Ballif, B. A., Smogorzewska, A., McDonald, E. R., Hurov, K. E., Luo, J., Bakalarski, C. E., Zhao, Z., Solimini, N., Lerenthal, Y., Shiloh, Y., Gygi, S. P., & Elledge, S. J. (2007). ATM and ATR substrate analysis reveals extensive protein networks responsive to DNA damage. Science, 316, 1160–1166. 10.1126/science.1140321 17525332

[acel13458-bib-0047] Mayr, B., & Montminy, M. (2001). Transcriptional regulation by the phosphorylation‐dependent factor CREB. Nature Reviews Molecular Cell Biology, 2, 599–609. 11483993 10.1038/35085068

[acel13458-bib-0048] Mehlen, P., Preville, X., Chareyron, P., Briolay, J., Klemenz, R., & Arrigo, A. P. (1995). Constitutive expression of human hsp27, Drosophila hsp27, or human alpha B‐crystallin confers resistance to TNF‐ and oxidative stress‐induced cytotoxicity in stably transfected murine L929 fibroblasts. The Journal of Immunology, 154, 363–374. 7995955

[acel13458-bib-0049] Merk, D. J., Ohli, J., Merk, N. D., Thatikonda, V., Morrissy, S., Schoof, M., Schmid, S. N., Harrison, L., Filser, S., Ahlfeld, J., Erkek, S., Raithatha, K., Andreska, T., Weisshaar, M., Launspach, M., Neumann, J. E., Shakarami, M., Plenker, D., Marra, M. A., … Schuller, U. (2018). Opposing effects of CREBBP mutations govern the phenotype of Rubinstein‐Taybi syndrome and adult SHH medulloblastoma. Developmental Cell, 44, 709–724. 10.1016/j.devcel.2018.02.012 29551561

[acel13458-bib-0050] Miura, T., Mattson, M. P., & Rao, M. S. (2004). Cellular lifespan and senescence signaling in embryonic stem cells. Aging Cell, 3, 333–343. 10.1111/j.1474-9728.2004.00134.x 15569350

[acel13458-bib-0051] Montminy, M. R., Sevarino, K. A., Wagner, J. A., Mandel, G., & Goodman, R. H. (1986). Identification of a cyclic‐AMP‐responsive element within the rat somatostatin gene. Proceedings of the National Academy of Sciences of the United States of America, 83, 6682–6686. 10.1073/pnas.83.18.6682 2875459 PMC386573

[acel13458-bib-0052] Moorhead, G. B., Trinkle‐Mulcahy, L., & Ulke‐Lemee, A. (2007). Emerging roles of nuclear protein phosphatases. Nature Reviews Molecular Cell Biology, 8, 234–244. 10.1038/nrm2126 17318227

[acel13458-bib-0053] Mumby, M. C., & Walter, G. (1993). Protein serine/threonine phosphatases: Structure, regulation, and functions in cell growth. Physiological Reviews, 73, 673–699. 8415923 10.1152/physrev.1993.73.4.673

[acel13458-bib-0054] Navarro, X., Geuna, S., Grothe, C., & Haastert‐Talini, K. (2018). Introduction: Thematic papers issue on peripheral nerve regeneration and repair. Anatomical Record, 301, 1614–1617. 10.1002/ar.23941 30299596

[acel13458-bib-0055] Olsen, J. V., Blagoev, B., Gnad, F., Macek, B., Kumar, C., Mortensen, P., & Mann, M. (2006). Global, in vivo, and site‐specific phosphorylation dynamics in signaling networks. Cell, 127, 635–648. 10.1016/j.cell.2006.09.026 17081983

[acel13458-bib-0056] Ousman, S. S., Tomooka, B. H., van Noort, J. M., Wawrousek, E. F., O'Connor, K. C., Hafler, D. A., Sobel, R. A., Robinson, W. H., & Steinman, L. (2007). Protective and therapeutic role for alphaB‐crystallin in autoimmune demyelination. Nature, 448, 474–479. 17568699 10.1038/nature05935

[acel13458-bib-0057] Pan, H., & Finkel, T. (2017). Key proteins and pathways that regulate lifespan. Journal of Biological Chemistry, 292, 6452–6460. 10.1074/jbc.R116.771915 PMC5399099 28264931

[acel13458-bib-0058] Pelicci, P. G. (2004). Do tumor‐suppressive mechanisms contribute to organism aging by inducing stem cell senescence? Journal of Clinical Investigation, 113, 4–7. 10.1172/JCI200420750 PMC300887 14702099

[acel13458-bib-0059] Qin, J., Chen, H. G., Yan, Q., Deng, M., Liu, J., Doerge, S., Ma, W., Dong, Z., & Li, D. W. (2008). Protein phosphatase‐2A is a target of epigallocatechin‐3‐gallate and modulates p53‐Bak apoptotic pathway. Cancer Research, 68, 4150–4162. 10.1158/0008-5472.CAN-08-0839 18519674

[acel13458-bib-0060] Quinlan, R. A., & Hogg, P. J. (2018). gamma‐Crystallin redox‐detox in the lens. Journal of Biological Chemistry, 293, 18010–18011. 10.1074/jbc.H118.006240 PMC6240857 30446601

[acel13458-bib-0061] Raghavan, C. T., Smuda, M., Smith, A. J., Howell, S., Smith, D. G., Singh, A., Gupta, P., Glomb, M. A., Wormstone, I. M., & Nagaraj, R. H. (2016). AGEs in human lens capsule promote the TGFbeta2‐mediated EMT of lens epithelial cells: Implications for age‐associated fibrosis. Aging Cell, 15, 465–476. 26853893 10.1111/acel.12450 PMC4854921

[acel13458-bib-0062] Rakete, S., & Nagaraj, R. H. (2016). Identification of kynoxazine, a novel fluorescent product of the reaction between 3‐hydroxykynurenine and erythrulose in the human lens, and its role in protein modification. Journal of Biological Chemistry, 291, 9596–9609. 10.1074/jbc.M116.716621 PMC4850297 26941078

[acel13458-bib-0063] Reddy, V. N., Giblin, F. J., Lin, L. R., Dang, L., Unakar, N. J., Musch, D. C., Boyle, D. L., Takemoto, L. J., Ho, Y. S., Knoernschild, T., Juenemann, A., & Lutjen‐Drecoll, E. (2001). Glutathione peroxidase‐1 deficiency leads to increased nuclear light scattering, membrane damage, and cataract formation in gene‐knockout mice. Investigative Ophthalmology & Visual Science, 42, 3247–3255. 11726630

[acel13458-bib-0064] Riccio, A., Ahn, S., Davenport, C. M., Blendy, J. A., & Ginty, D. D. (1999). Mediation by a CREB family transcription factor of NGF‐dependent survival of sympathetic neurons. Science, 286, 2358–2361. 10.1126/science.286.5448.2358 10600750

[acel13458-bib-0065] Sakaguchi, K., Herrera, J. E., Saito, S., Miki, T., Bustin, M., Vassilev, A., Anderson, C. W., & Appella, E. (1998). DNA damage activates p53 through a phosphorylation‐acetylation cascade. Genes & Development, 12, 2831–2841. 10.1101/gad.12.18.2831 9744860 PMC317174

[acel13458-bib-0066] Schey, K. L., Wang, Z., Friedrich, M. G., Garland, D. L., & Truscott, R. (2020). Spatiotemporal changes in the human lens proteome: Critical insights into long‐lived proteins. Progress in Retinal and Eye Research, 76, 100802. 10.1016/j.preteyeres.2019.100802 31704338 PMC7328127

[acel13458-bib-0067] Sharpless, N. E., & DePinho, R. A. (2004). Telomeres, stem cells, senescence, and cancer. Journal of Clinical Investigation, 113, 160–168. 10.1172/JCI20761 PMC311439 14722605

[acel13458-bib-0068] Sharma, K. K. (2016). Crystallin biochemistry in health and disease. Biochimica et Biophysica Acta, 1860, 147–148. 10.1016/j.bbagen.2015.10.018 26515633

[acel13458-bib-0069] Shiels, A., & Hejtmancik, J. F. (2019). Biology of inherited cataracts and opportunities for treatment. Annual Review of Vision Science, 5, 123–149. 10.1146/annurev-vision-091517-034346 PMC6791712 31525139

[acel13458-bib-0070] Shu, D. Y., & Lovicu, F. J. (2017). Myofibroblast transdifferentiation: The dark force in ocular wound healing and fibrosis. Progress in Retinal and Eye Research, 60, 44–65. 28807717 10.1016/j.preteyeres.2017.08.001 PMC5600870

[acel13458-bib-0071] Sperka, T., Wang, J., & Rudolph, K. L. (2012). DNA damage checkpoints in stem cells, ageing and cancer. Nature Reviews Molecular Cell Biology, 13, 579–590. 10.1038/nrm3420 22914294

[acel13458-bib-0072] Stewart, S. A., & Weinberg, R. A. (2006). Telomeres: Cancer to human aging. Annual Review of Cell and Developmental Biology, 22, 531–557. 10.1146/annurev.cellbio.22.010305.104518 16824017

[acel13458-bib-0073] Su, S. P., Lyons, B., Friedrich, M., McArthur, J. D., Song, X., Xavier, D., Truscott, R. J., & Aquilina, J. A. (2012). Molecular signatures of long‐lived proteins: Autolytic cleavage adjacent to serine residues. Aging Cell, 11, 1125–1127. 22805275 10.1111/j.1474-9726.2012.00860.x

[acel13458-bib-0074] Truscott, R. J., & Friedrich, M. G. (2016). The etiology of human age‐related cataract. Proteins don't last forever. Biochimica et Biophysica Acta, 1860, 192–198. 26318017 10.1016/j.bbagen.2015.08.016 PMC5489112

[acel13458-bib-0075] Wadzinski, B. E., Wheat, W. H., Jaspers, S., Peruski, L. J., Lickteig, R. L., Johnson, G. L., & Klemm, D. J. (1993). Nuclear protein phosphatase 2A dephosphorylates protein kinase A‐phosphorylated CREB and regulates CREB transcriptional stimulation. Molecular and Cellular Biology, 13, 2822–2834. 10.1128/MCB.13.5.2822 8386317 PMC359667

[acel13458-bib-0076] Wang, B., Hom, G., Zhou, S., Guo, M., Li, B., Yang, J., Monnier, V. M., & Fan, X. (2017). The oxidized thiol proteome in aging and cataractous mouse and human lens revealed by ICAT labeling. Aging Cell, 16, 244–261. 10.1111/acel.12548 28177569 PMC5334568

[acel13458-bib-0077] Wang, J., Lou, S. S., Wang, T., Wu, R. J., Li, G., Zhao, M., Lu, B., Li, Y. Y., Zhang, J., Cheng, X., Shen, Y., Wang, X., Zhu, Z. C., Li, M. J., Takumi, T., Yang, H., Yu, X., Liao, L., & Xiong, Z. Q. (2019). UBE3A‐mediated PTPA ubiquitination and degradation regulate PP2A activity and dendritic spine morphology. Proceedings of the National Academy of Sciences of the United States of America, 116, 12500–12505. 10.1073/pnas.1820131116 31160454 PMC6589679

[acel13458-bib-0078] Wang, L., Nie, Q., Gao, M., Yang, L., Xiang, J. W., Xiao, Y., Liu, F. Y., Gong, X. D., Fu, J. L., Wang, Y., Nguyen, Q. D., Liu, Y., Liu, M., & Li, D. W. (2020). The transcription factor CREB acts as an important regulator mediating oxidative stress‐induced apoptosis by suppressing alphaB‐crystallin expression. Aging, 12, 13594–13617. 32554860 10.18632/aging.103474 PMC7377838

[acel13458-bib-0079] Wang, Z., Friedrich, M. G., Truscott, R., & Schey, K. L. (2019). Cleavage C‐terminal to Asp leads to covalent crosslinking of long‐lived human proteins. Biochimica et Biophysica Acta (BBA) ‐ Proteins and Proteomics, 1867, 831–839. 10.1016/j.bbapap.2019.06.009 31226490 PMC9227964

[acel13458-bib-0080] Wang, Z., Lyons, B., Truscott, R. J., & Schey, K. L. (2014). Human protein aging: Modification and crosslinking through dehydroalanine and dehydrobutyrine intermediates. Aging Cell, 13, 226–234. 10.1111/acel.12164 24134651 PMC4114717

[acel13458-bib-0081] Wormstone, I. M., Wormstone, Y. M., Smith, A., & Eldred, J. A. (2020). Posterior capsule opacification: What's in the bag? Progress in Retinal and Eye Research, 82, 100905. 32977000 10.1016/j.preteyeres.2020.100905

[acel13458-bib-0082] Xia, Z., Dickens, M., Raingeaud, J., Davis, R. J., & Greenberg, M. E. (1995). Opposing effects of ERK and JNK‐p38 MAP kinases on apoptosis. Science, 270, 1326–1331. 10.1126/science.270.5240.1326 7481820

[acel13458-bib-0083] Xiao, L., Gong, L. L., Yuan, D., Deng, M., Zeng, X. M., Chen, L. L., Zhang, L., Yan, Q., Liu, J. P., Hu, X. H., Sun, S. M., Liu, J., Ma, H. L., Zheng, C. B., Fu, H., Chen, P. C., Zhao, J. Q., Xie, S. S., Zou, L. J., … Li, D. W. (2010). Protein phosphatase‐1 regulates Akt1 signal transduction pathway to control gene expression, cell survival and differentiation. Cell Death and Differentiation, 17, 1448–1462. 10.1038/cdd.2010.16 20186153

[acel13458-bib-0084] Yin, J. C., Del, V. M., Zhou, H., & Tully, T. (1995). CREB as a memory modulator: Induced expression of a dCREB2 activator isoform enhances long‐term memory in Drosophila. Cell, 81, 107–115. 10.1016/0092-8674(95)90375-5 7720066

[acel13458-bib-0085] Yin, J. C., Wallach, J. S., Del, V. M., Wilder, E. L., Zhou, H., Quinn, W. G., & Tully, T. (1994). Induction of a dominant negative CREB transgene specifically blocks long‐term memory in Drosophila. Cell, 79, 49–58. 10.1016/0092-8674(94)90399-9 7923376

[acel13458-bib-0086] Zhou, B. B., & Elledge, S. J. (2000). The DNA damage response: Putting checkpoints in perspective. Nature, 408, 433–439. 11100718 10.1038/35044005

