# Supplementary material for: PP‐1β and PP‐2Aα modulate cAMP response element‐binding protein (CREB) functions in aging control and stress response through de‐regulation of αB‐crystallin gene and p300‐p53 signaling axis
Source: Aging Cell. 2021 Aug 23;20(9):e13458. doi: 10.1111/acel.13458 (PMC8441381; doi:10.1111/acel.13458)
Supplement: Supplementary file 1 — Supplementary Material [file ACEL-20-e13458-s001.pdf]

Supplementary Data

1. Appendix S1

1.1 Chemicals

Various molecular biology reagents were purchased from Invitrogen Life Technologies, Gaithersburg, MD; Stratagene, La Jolla, CA; and Promega Biotech, Madison, WI. All the oligos, DNA and protein size markers were purchased from Invitrogen Life Technologies, Gaithersburg, MD; Dongsheng Biotech. Co. Ltd; Guangzhou; and Genstar Biotech. Inc., Beijing, China. Various antibodies were obtained from Cell Signaling Technology, Boston, MA; abCam Inc., Cambridge, MA; Santa Cruz Biotechnology, Inc. Dallas, TX; Tingke Biological Technology, Inc., Beijing; and the Proteintech Group, Inc., Beijing, China. The culture medium, and most other chemicals and antibiotics were purchased from Sigma-Aldrich, St. Louis, MO and Invitrogen Life Technologies, Gaithersburg, MD.

1.2 Collection of human lens capsular epithelial samples

The collection of human capsular epithelia from cataract lenses of different age groups was approved by the Institutional Review Board of the Zhongshan Ophthalmic Center (ZOC). Informed consent was obtained from each of the cataract patients. For senile cataractous samples, the lens capsules from cataract patients were collected at surgery by the physicians in Zhongshan Ophthalmic Center of Sun Yat-sen University. According to the patient age, capsular samples from 50 to 59 years old were grouped together and labeled as 50s (Table S3); those from 60 to 69 years old were grouped together and labeled as 60s (Table S4); those from 70 to 79 years old were

grouped together and labeled as 70s (Table S5), and those from 80 to 89 years old were grouped together and labeled as 80s (Table S6). As control, the capsular samples from the lenses of human donors were listed in Table S2.

1.3 CRISPR/Cas9-mediated gene editing to generate S133A-CREB mouse model

The CREB-S133A mutant heterozygote mouse model was generated by co-injection of Cas9 mRNA, short guide RNA (sgRNA) and donor CREB(S133A) oligo (Fig.6a). In short, super-ovulated female C57BL/6 mice were mated to male C57BL/6 mice, and embryos were collected from oviducts. Cas9 mRNA, sgRNAs targeting the CREB exon5 gene locus as well as donor CREB(S133A) oligo were co-injected into the pronuclei of one-cell embryos. The injected embryos were cultured in vitro for two hours, then the survived embryos were transplanted into pseudo-pregnant mice. At 1 week after birth, genomic DNA from the toes of the newborn F0 mice was extracted for PCR analysis and DNA sequencing.

1.4 Culture of mouse lens epithelial cells (αTN4-1) and mouse skin epithelial cells (JB6) and their treatment by okadaic acid (OA) or LB100 treatment

The mouse lens epithelial cells, αTN4-1, were kindly provided by Dr. Paul Russel of the National Eye Institute, and grown in Dulbecco's Modified Eagle's Medium (D7777, Sigma; and 11995500, Gibco Inc., Beijing) containing 10% fetal bovine serum as described previously (Li et al., 2006). The mouse skin epithelial cells, JB6 was kindly provided by Dr. Zigang Dong of the Hormel Institute of the University of Minnesota, and grown in the conditions as described before (Qin et al., 2008). For the okadaic acid (OA) treatment, the αTN4-1 and JB6 cells were grown to 90% confluence in DMEM containing 10% fetal bovine serum. Then, the media containing 10% serum

plus 0.1% DMSO (control) or 20 to 200 nM okadaic acid (experiment) were used to replace the culture media for an incubation of additional 3 hours. For the LB100 treatment, the αTN4-1 cells were grown to 90% confluence in DMEM containing 10% fetal bovine serum. Then, the media containing 10% serum plus 0 to 16 μM LB100 were used to replace the culture media for an incubation of 3 hours, then harvested for analysis.

1.5 Silence of PP-1 or PP-2A subunits and overexpression of PP-1β and PP-2Acα in αTN4-1 cells

The stable constructs for silencing various isoforms of PP-1 and PP-2A catalytic subunits and the Tet-on system were described before (Li et al., 2006; Xiao et al., 2010). Different plasmids generating specific shRNAs for each isoform of PP-1 and PP-2A catalytic subunits were obtained from Open Biosystems Inc (Xiao et al., 2010) and subsequently amplified at the laboratory. These amplified constructs were then transfected into αTN4-1 cells using lipofectamine 2000™ according to the instruction, and stable clones expressing shRNA for each catalytic subunit of PP-1 and PP-2A were established under screening by puromycin (1μg/ml) for 4–6 weeks. The stable clones were verified by western blot analysis.

For overexpression vectors, the construction of full-length cDNA of PP-1β was cloned by PCR. These cDNA were subcloned into pCI-Neo vector at the Xho I/Sal I. The primers used for PP-1β are listed in Table S7. The pCI-PP-2Acα overexpression construct was described before (Qin et al., 2009).

1.6 Establishment of stable expression cell lines

The CREB and S133A-CREB were cloned into pCI-Neo vector at EcoRI and XbaI sites and amplified in DH-5α purified by two rounds of CsCl ultracentrifugation as previously described (Mao et al., 2004; Li et al., 2005; 2006). Transfection of αTN4-1 cells was performed using Lipofectamine 2000^{TM} from the Invitrogen Life Technologies according to the company instruction manual. The cells transfected with pCI-Neo, pCI-CREB, and pCI-S133A-CREB were then subjected to G418 (500 μg/ml) selection for 4-6 weeks and subsequently individual clones for the following stable line were established. These include pCI-Neo-αTN4-1, pCI-CREB-αTN4-1, and pCI-S133A-CREB-αTN4-1 cells.

1.7 RNA interference and lentivirus infection

For shRNA-mediated gene knockdown, a set of single-stranded oligonucleotides encoding the mock or CREB 3’UTR target shRNA and its complement were synthesized. CREB 3′ UTR shRNA: sense, 5’-CCGGGCCTGAAAGCAACTACAGAATCTCGAGATTCTGTAGTTGCTTTCAGGCTTTTTG-3’, anti-sense, 5’-AATTCAAAAAGCCTGAAAGCAACTACAGAATCTCGAGATTCTGTAGTTGCTTTCAGGC-3’. Control shRNA：sense，5′-CCGGAAGCTGGAGTACAACTACAACCTCGAGGTTGTAGTTGTACTCCAGCTTTTTTT-G3′，anti-sense，5′-AATTCAAAAAAAGCTGGAGTACAACTACAACCTCGAGGTTGTAGTTGTACTCCAGCTT-3′. The oligonucleotide sense and antisense pair were annealed and inserted into pLKO lentiviral expression system.

For the lentivirus infections, the psPAX2 packaging plasmid, pMD2.G envelope plasmid and the pLKO-NC shRNA, pLKO- CREB 3′ UTR shRNA constructs were used to co-transfect HEK-293FT cells. Virus supernatant was collected 48 h post-transfection, filtered through a 0.45 mm

polyethersulfone filter. The virus suspension was mixed with 8 μg/ml polybrene to infect αTN4-1, pCI-Neo-αTN4-1， pCI-CREB-αTN4-1 and pCI-S133A-CREB-αTN4-1 cells. Puromycin screening was conducted 48 h post-infection. Western blot analyses were used to confirm gene knockdown.

1. 8 Treatment by 40 mU glucose oxidase

The parent αTN4-1 cells, pCI-Neo-αTN4-1 cells, pCI-CREB-αTN4-1 and pCI-S133A-CREB-αTN4-1 (transfected without shCREB-3'UTR, Figs.3, Fig.4a, Fig.S1, S2 and S3; or transfected with shCREB-3'UTR, Fig.4b-4c, Fig.5a-5f, Fig.5g-shCREB and Fig.S4) cells were grown to 90% confluence in DMEM containing 10% fetal bovine serum. Then, the serum-free media with 40mU glucose oxidase (GO) were used to replace the culture media for the required period of incubation as indicated. After treatment, all samples were collected for analysis of apoptosis and gene expression. Under 40 mU GO treatment, the production of H_2O_2 and decrease in protein thiol were determined as previously reported (Gong et al., 2018; Wang et al. 2020; Sun et al., 2020).

1.9 Apoptosis analysis with cellTiter-Glo® luminescent cell viability assay and live/dead viability/cytotoxicity

The percentage of apoptotic cells was determined either by cellTiter-Glo® luminescent cell viability assay kit (Promega, G7573) or using live/dead viability/ cytotoxicity kit (Thermofisher Scientific, L3224) as previously described (Wang et al., 2020; Nie et al., 2021). The CellTiterGlo® Luminescent Cell Viability Assay is a homogeneous method to determine the number of viable cells in culture based on quantification of the ATP present which signals the presence of metabolically active cells. About $2x10^4$ cells were seeded into each well of 96-well

plates, 12h later, the culture media were replaced with 100ul medium containing 40 mU GO to induce cell apoptosis. After treatment, the same volumes of the mixed CellTiter-Glo® Buffer and CellTiter-Glo® Substrate were added into each well and luminescence was read by synergy microplate reader (BioTek).

1.10 Total protein extraction and western blot analysis

For cultured cells, total proteins were prepared from 0 to 200nM OA treated αTN4-1 and JB6 cells, 0 to 16 μM LB100-treated αTN4-1 cells, mock or 40 mU GO-treated αTN4-1, pCI-Neo-αTN4-1, pCI-CREB-αTN4-1 or pCI-S133A-CREB-αTN4-1(transfected without shCREB-3'UTR, Figs.3, Fig.4a, Fig.S1, S2 and S3; or transfected with shCREB-3'UTR, Fig.4b-4c, Fig.5a-5f, Fig.5g-shCREB and Fig.S4) cells for 0 to 6 hours. After treatment, total proteins were extracted using RIPA buffer (50 mM Tris·HCl (pH7.4), 150 mM NaCl, 2 mM EDTA, 1% NP-40, 0.1% SDS, 1% sodium deoxycholate) in the presence of the protease inhibitor cocktail. After homogenization by passing through an initial 18.5-gauge needle followed by the 23.5G needle, the cell lysate was centrifuged at 10,000 x g for 20 min at 4°C, the supernatant fraction of each sample was collected and stored in aliquots at –80°C. Twenty to one hundred micrograms of total proteins in each sample were resolved by 8%, 10 % or 12% SDS-polyacrylamide gel and transferred into PVDF membranes (Li et al., 1995; Li and Spector, 1996; Li et al, 2005; Yan et al., 2007 and 2010; Gong et al., 2014 and 2018; Wang et al., 2020; Gong et al., 2021). For capsular samples of lens epithelium, each capsular sample from 6 controls or 66 cataractous lenses of different ages (see Table S2-S6 for details), was transferred to an Eppendorf tube containing 50 μl RIPA buffer and homogenized on ice with an Eppendorf tube micro pestle (Brinkmann Instruments Inc.). For each sample, the protein concentration was determined as previously described (Li et al., 2006; Xiao et

al., 2010; Liu et al. 2020) and analyzed using AWI (see below). For regular western blot analysis, the protein blots were blocked with 5% non-fat milk in TBST (10 mM Tris HCl/pH8.0, 150 mM NaCl, 0.05% Tween-20) for 1 h at room temperature. Each membrane was then incubated overnight at 4°C with following primary antibodies: anti-PP-1α (sc-271762), anti-PP-1β (sc-6107) , anti-PP1γ (sc-6108), anti-PP-2Acα (sc-32401) and anti-PP-2Acβ (sc-80665) from Santa Cruz Biotechnology; anti-CREB (CST-4820), anti-p-CREB-S133 (CST-9198) anti-p300 (CST-70088), anti-Pcaf (CST-3378), anti-p53(CST-2524), anti-acetyl-p53-k379/382 (mouse/human; CST-2570), and anti-Bak (CST-12105) antibodies from Cell Signaling Inc.; anti-Bax (ab 32503) and anti-PP2Aα (ab106262) from Abcam Inc.; and anti-αB-crystallin antibody (generous gift of Dr. J Horwitz in the Julie Eye Institute of UCLA), and anti-β-actin (66009), anti-GAPDH (60004), anti-α-actinin (113-2-AP) as well as anti-β-tubulin antibodies (66240) from the Proteintech Group, Inc. at a dilution of 1 to 500 to 5,000 (μg/ml) in 5% milk prepared in TBS (for total proteins) or 5% BSA in TBS (for phosphor-antibody). After three 10-minute washes with TBST, each blot was incubated with the HRP-conjugated secondary antibody (anti-mouse and rabbit IgG from CST) diluted at 1:1000 or 2000 in blocking solution at room temperature for 1 h. The blots were visualized using a Tanon chemiluminescence system (China).

1.11 Automated western immunoblotting (AWI)

The simple western immunoblots were performed on a Wes (ProteinSimple) as previously described (Dahl et al. 2016; Liu et al. 2020). Briefly, each sample was loaded with 0.9 μg total protein and then analyzed with the Size Separation Master Kit and Split Buffer (12-230 kDa) according to the manufacturer's standard instruction using the antibodies described above. The

dilution factors are 1:100 for CREB, p53, p53-K379/K382, Pcaf, PP1β, PP2Aα, β-Actin and 1:1000 for αB-crystallin. The Campass software (Protein Simple, version 4.1.5) was used to program the Wes and for presentation (and quantification) of the western blots. Output western blot style data were displayed with exposure time indicated, and the quantification data were from the software-calculated average of seven exposures (1-512 s).

1.12 RT-PCR, qRT-PCR and RNAseq analysis

RT-PCR and qPCR were conducted as we described previously (Wang et al. 2020). Total RNAs were extracted using the TRIzol Reagent (Invitrogen). cDNA synthesis was performed with 1 μg of total RNAs using the HiScript II Q RT SuperMix for qPCR (+gDNA wiper) kit (Vazyme, R223-01). Gene expression levels were analyzed using ChamQ SYBR Color qPCR Master Mix (Vazyme, Q411-02) and the LightCycler 480 qPCR system (Roche). The assays were performed in triplicate, and the Ct values were normalized to β-actin. All the primers used for this study are listed in Table S7.

For the RNAseq analyses, total RNAs were extracted from pCI-CREB-αTN4-1 and pCI-S133A-CREB-αTN4-1cells using the TRIzol reagent according to the manufacturer's instruction. Preparation of the RNAseq library and subsequent sequencing were conducted by the Berry Genomics Corporation. Pooled samples of two biological repeats were sequenced on Illumina Nova 6000. The obtained sequence reads were cleaned and mapped to (GRCm38/mm10) using Tophat. Gene expression and changes were analyzed using Bowtie2 and RSEM. The relative abundance of mRNAs was normalized and presented as fragments per kilobase of transcript per million mapped reads (FPKM). Hierarchical cluster and scatter plot analyses of gene expression levels were performed using the R software (http://www.r-project.org/). KEGG analysis was

carried out by Kobas. Samples harvested from two independent experiments were pooled and used for each RNAseq analysis.

1.13 Establishment of Bak, Bax and Bak/Bax knockout stable cell lines

The CRISPR/Cas9 system contains Px459 pSpCas9(BB)-2A-Puro-MSC vector and two hU6-gRNAs. The sgRNAs targeting Bak and Bax loci were designed through the website http://chopchop.cbu.uib.no/. Two sgRNAs were designed to target Bak or Bax locus, respectively. The primers for targeting Bak locus include 5'-CAAGTTGTCCATCTCGGGGTTGG-3' (target 1) and 5' TCTTCACCAAGATCGCCTCCAGG 3' (target 2), and the primers targeting Bax locus include 5'-CCAGTTCATCTCCAATTCGCCGG-3' (target 1) and 5'-CCCCCGAGAGGTCTTCTTCCGG-3' (target 2). The synthesized oligonucleotide pairs for sgRNAs (Table S7) were annealed and cloned into Px459 pSpCas9(BB)-2A-Puro-MSC (target 1, gRNA) and EZ-GuideXH (target 2, gRNA) vectors separately using the restriction enzyme BbsI. EZ-GuideXH is an auxiliary vector for insertion of a second guide RNA. The synthesized oligonucleotide pairs inserted Px459 pSpCas9(BB)-2A-Puro-MSC and EZ-GuideXH were digested by XhoI and HindIII. After digestion, the short fragments of EZ-GuideXH containing the hU6-gRNAs for target 2 locus were cloned into Px459 pSpCas9(BB)-2A-Puro-MSC which has hU6-gRNAs for target 1 locus. The CRISPR/Cas9 plasmids were amplified with stbl3 and transfect into pCI-CREB-αTN4-1 cell with lipofectamine 3000™ according to the instruction. And the knockout stable clones were established under screening by puromycin (1μg/ml) for 4 weeks. The stable clones were verified by western blot and DNA sequencing.

1.14 DNA sequencing

To further verify the stable Bak, Bax and Bak/Bax knockout clones (which were first verified by western blot), we first isolated the genomic DNA from the above 3 clones using TIANamp Genomic DNA Kit (DP304, TIANGEN BIOLOTECH Co., LTD, Beijing). The edited DNA fragments of Bak and Bax genes through CRISPR/Cas9 were amplified with following primers: 5'- GGACAGCTTGCCTTTGCTGA-3'(Forward) and 5'- CAGGGACACTGACTTCCGGT-3'(Reverse) for Bak gene and 5'-GACCTTGGAGCAGCCGC-3'(Forward) and 5'-TTCCCTGGTCCTCACAGACC-3' (Reverse) for Bax gene. The amplified Bak or Bax gene fragments were ligated into pMD20-T vector through Mighty TA-cloning Kit (Takara, 6028) according to the instruction and the positive plasmids containing the amplified Bak or Bax gene fragments were further amplified with DH5α, and then sequenced by Tsingke Biological Technology, Inc.

1.15 Statistical analysis

All experiments were repeated at least three times (N=3) except for RNAseq analysis in which each analyzed sample was a pool of two separated samples (N=4). Significance was determined by two-tailed Student's t-test. The error bar in all figures represents standard deviation.

1.16 References for Experimental Procedures

Gong L, Fangyuan Liu, Zhen Xiong, Ruili Qi, Zhongwen Luo, Xiaodong Gong, Qian Nie, Qian Sun, Yunfei Liu, Wenjie Qing, Ling Wang, Lan Zhang, Xiangcheng Tang, Shan Huang, Ling Wang, Gen Li, Hong Ouyang, Mengqing Xiang, Quan Dong Nguyen,Yizhi Liu and David Wan-Cheng Li (2018) Heterochromatin protects retinal pigment epithelium cells from oxidative damage by silencing p53 target genes. Proc. Natl. Acad. Sci. USA **115,** E3987-E3995.

Gong L, Weike Ji, Xiao-Hui Hu,Wen-Feng Hu, Xiang-Cheng Tang, Zhao-Xia Huang, Ling Li, Mugen Liu, Shihua Xiang, Erxi Wu, Zachary Woodward, Yizhi Liu, Quan Dong Nguyen, and David Wan-Cheng Li (2014) Sumoylation differentially regulates Sp1 to control cell differentiation. Proc. Natl. Acad. Sci. USA **111,** 5574-

5579.

Gong XD, Wang Y, Hu XB, Zheng SY, Fu JL, Nie Q, Wang L, Hou M, Xiang JW, Xiao Y, Gao Q, Bai YY, Liu Y and Li. DW (2021). Aging-Dependent Loss of GAP Junction Proteins Cx46 and Cx50 in the Fiber Cells of Human and Mouse Lenses Accounts for the Diminished Coupling Conductance. Aging (Albany NY). 13, In Press.

Li DW, Liu JP, Mao YW, Xiang H, Wang J, Ma WY (2005) Calcium-activated RAF/MEK/ERK signaling pathway mediates p53-dependent apoptosis and is abrogated by alpha B-crystallin through inhibition of RAS activation. Mol Biol Cell 2005, **16,** 4437-4453.

Li DW, Liu JP, Schmid PC, Schlosser R, Feng H, Liu WB (2006) Protein serine/threonine phosphatase-1 dephosphorylates p53 at Ser-15 and Ser-37 to modulate its transcriptional and apoptotic activities. Oncogene **25**, 3006-3022.

Li WC, Kuszak JR, Dunn K, Wang RR, Ma WC, Wang GM, Spector A, Leib M, Cotliar AM, Weiss M, Espy J, Howard G, Farris RL, Auran J, Donn A, Hofeldt A, Mackay C, Merriam J, Mittl R, Smith TR. (1995). Lens epithelial cell apoptosis appears to be a common cellular basis for non-congenital cataract development in humans and animals. J. Cell Biol. **130**, 169–181.

Li WC, Spector A. (1996). Lens Epithelial cell apoptosis is an early event in the development of UVB-induced cataract. Free Radic. Biol. Med**.** **20**, 301–311.

Liu FY, Fu JL, Wang L, Nie Q, Luo ZW, Hou M, Yang Y, Gong XD, Wang Y, Xiao Y, Xiang JW, Hu XB, Zhang L, Wu MX, Chen WR, Cheng B, Luo LX, Zhang XY Liu XL, Zheng DY, Huang S, Liu YZ, Li DW (2020). Molecular Signature for Senile and Complicated Cataracts Derived from Analysis of Sumoylation Enzymes and their Substrates in Human Cataract Lenses. Aging Cell. **19**, e13222: 10.1111/acel.13222. Epub ahead of print. PMID:32827359.

Mao YW, Liu JP, Xiang H, Li DW (2004). Human alphaA- and alphaB-crystallins bind to Bax and Bcl-X(S) to sequester their translocation during staurosporine-induced apoptosis. Cell Death Differ. **11**, 512-26.

Nie Q, Chen H, Zou M, Wang L, Hou M, Xiang J-W, Luo Z, Gong X-D, Fu J-L, Wang Y, Zheng S-Y, Xiao Y, Gan Y-W, Gao Q, Bai Y-Y, Wang J-M, Zhang L, Tang X-C, Hu X, Gong L, Liu Y and Li DW (2021). The E3 Ligase PIAS1 Regulates p53 Sumoylation to Control Stress-Induced Apoptosis of Lens Epithelial Cells through the Proapoptotic Regulator Bax. Frontiers in Cell & Dev. Biol. 9:660494. Doi:10.3389/fcell.2021.660494.

Qin J, Chen HG, Yan Q, Deng M, Liu J, Doerge S, Ma W, Dong Z, Li DW (2008). Protein phosphatase-2A is a target of epigallocatechin-3-gallate and modulates p53-Bak apoptotic pathway. Cancer Res. **68**, 4150-62.

Sun Q, Gong L, Qi R, Qing W, Zou M, Ke Q, Zhang L, Tang X, Nie Q, Yang Y, Hu A, Ding X, Lu L, Liu Y, Li DW (2020). Oxidative stress-induced KLF4 activates inflammatory response through IL17RA and its downstream targets in retinal pigment epithelial cells. Free Radic Biol Med. **147**, 271-281.

Xiao L, Gong LL, Yuan D, Deng M, Zeng XM, Chen LL (2010) Protein phosphatase-1 regulates Akt1 signal transduction pathway to control gene expression, cell survival and differentiation. Cell Death Differ. **17,** 1448-1462.

Yan Q, Gong L, Deng M, Zhang L, Sun S, Liu J, Ma H, Yuan D, Chen PC, Hu X, Liu J, Qin J, Xiao L, Huang XQ, Zhang J, Li DW. (2010). Sumoylation activates the transcriptional activity of Pax-6, an important transcription factor for eye and brain development. Proc. Natl. Acad. Sci. USA **107**, 21034 9.

Yan Q, Liu WB, Qin J, Liu J, Chen HG (2007) Protein phosphatase-1 modulates the function of Pax-6, a transcription factor controlling brain and eye development. J. Biol. Chem. 282, 13954-13965.

Wang L, Nie Q, Gao M, Yang L, Xiang JW, Xiao Y, Liu FY, Gong XD, Fu JL, Wang Y, Nguyen QD, Liu Y, Liu M, Li DW. (2020). The transcription factor CREB acts as an important regulator mediating oxidative stress-induced apoptosis by suppressing αB-crystallin expression. Aging (Albany NY). 12 (13):13594-13617.

2. Appendix 2

Table S1. Summary of Age-Dependent Changes in PP-1β, PP-2Aα and CREB-Regulated Target Molecules in Normal and Cataract Samples of Different Age Groups[a]

CREB its Regulated Targets	30s Control	50s Control	60s Control	50s[b]	60s[b]	70s[b]	80s[b]
**PP-1β**	+++++	N/A	++++++	+++	++++	++++	++++
**PP-2Aα**	++++	N/A	++++++	+++++	+++++	+++++	+++++
**CREB**	++++++	++++++	+++++	++++++	+++++++	++++++	+++++++
**αB-Crystallin**	++++++	++++++	++++++	+++++	+++++	++++	++++
**p53**	++++++	++++++	+++++++	++++++	++++++	++++++	++++++
**P53-K379-Acetyl**	++	++	+	++	+++	++	++
**Pcaf**	+++	N/A	++	+++	+++	+++	+++

a. The summary data are derived from AWI analysis of limited normal lenses (Table S1) and cataract capsular epithelial samples (Table S2 to Table S5).

b. The levels of all samples from the patients were given from AWI averaged data of different exposure time. The units given represent absolute amount of the specific protein in 0.9 μg total protein samples. +++++++: > 5 x10^5 units; 5 x 10^5 > ++++++ > or = 2 x 10^5 units; 2 x 10^5 > +++++ > or =1 x 10^5 units; 1 x 10^5 units > ++++ > or = 5 x 10^4 units; 5 x 10^4 units > +++ > or =1 x 10^4 units; 1 x 10^4 units > ++ > or =5 x 10^3 units; and 5 x 10^3 units > + > or =1 x 10^3 units.

c. N/A samples were used up, not available as control for these studies.

Table S2. Donor Information of Normal Human Lenses Used in the Automated Wes Analysis

Number	Gender	Age	Lens Tissue Used
D1	Male	35	Lens Epithelium
D2	Female	45	Lens Epithelium
D3	Female	56	Lens Epithelium
D4	Male	61	Lens Epithelium
D5	Male	64	Lens Epithelium
D6	Male	65	Lens Epithelium

Table S3. Senile Cataract Patient Information of 50s (50-59 years old) Used in the Automated Wes Analysis

Number	Gender	Age	Diagnosed subtype
P1	Male	50	Complicated Cataract
P2	Female	50	Complicated Cataract
P3	Female	52	Nuclear Cataract
P4	Male	53	Nuclear Cataract
P5	Male	54	Cortical Cataract
P6	Female	54	NuclearCataract
P7	Female	56	Nuclear Cataract
P8	Female	56	Cortical Cataract
P9	Male	56	Nuclear Cataract
P10	Male	57	Nuclear Cataract
P11	Male	58	Complicated Cataract
P12	Female	59	Nuclear Cataract

Table S4. Senile Cataract Patient Information of 60s (60-67 years old) Used in the Automated Wes Analysis

Number	Gender	Age	Diagnosed subtype
P1	Female	60	Nuclear Cataract
P2	Female	61	Nuclear Cataract
P3	Female	61	Nuclear Cataract
P4	Male	61	Cortical Cataract
P5	Male	62	Cortical Cataract
P6	Female	62	Nuclear Cataract
P7	Male	63	Nuclear Cataract
P8	Male	63	Nuclear Cataract
P9	Male	63	Nuclear Cataract
P10	Female	63	Nuclear Cataract
P11	Female	63	Nuclear Cataract
P12	Male	64	Nuclear Cataract
P13	Male	65	Cortical Cataract
P14	Male	66	Cortical Cataract
P15	Female	66	Nuclear Cataract
P16	Female	66	Nuclear Cataract
P17	Female	66	Nuclear Cataract
P18	Female	67	Nuclear Cataract

Table S5. Senile Cataract Patient Information of 70s (70-77 years old) Used in the Automated Wes Analysis

Number	Gender	Age	Diagnosed subtype
P1	Male	70	Nuclear Cataract
P2	Female	70	Nuclear Cataract
P3	Male	71	Nuclear Cataract
P4	Female	71	Nuclear Cataract
P5	Female	71	Nuclear Cataract
P6	Female	71	Nuclear Cataract
P7	Female	71	Cortical Cataract
P8	Male	74	Nuclear Cataract
P9	Male	74	Nuclear Cataract
P10	Female	74	Nuclear Cataract
P11	Female	74	Nuclear Cataract
P12	Male	75	Nuclear Cataract
P13	Male	75	Nuclear Cataract
P14	Male	75	Nuclear Cataract
P15	Male	76	Nuclear Cataract
P16	Female	76	Nuclear Cataract
P17	Female	76	Nuclear Cataract
P18	Male	77	Nuclear Cataract

Table S6. Senile Cataract Patient Information of 80s (80-86 years old) Used in the Automated Wes Analysis

Number	Gender	Age	Diagnosed subtype
P1	Male	80	Cortical Cataract
P2	Female	80	Cortical Cataract
P3	Female	80	Cortical Cataract
P4	Female	80	Nuclear Cataract
P5	Female	80	Nuclear Cataract
P6	Male	81	Nuclear Cataract
P7	Male	81	Cortical Cataract
P8	Female	81	Nuclear Cataract
P9	Female	81	Nuclear Cataract
P10	Female	81	Nuclear Cataract
P11	Male	82	Nuclear Cataract
P12	Male	84	Nuclear Cataract
P13	Male	85	Nuclear Cataract
P14	Male	85	Nuclear Cataract
P15	Female	85	Nuclear Cataract
P16	Female	85	Cortical Cataract
P17	Female	85	Nuclear Cataract
P18	Male	86	Cortical Cataract

Table S7. Oligo Primers Used in QRT-PCR and Gene Knockout, Gene Knockdown or Overexpression

Specific Gene	Primer Directions	Primer Sequences
Mouse Bak	F	ACCGGCGCTACGACACAGAG
	R	TAGGCTGGAGGCGATCTTGGTG
Mouse Bax	F	AGGATGCGTCCACCAAG
	R	AAAGTAGAAGAGGGCAACCA
Mouse Bcl2	F	CTACCGTCGTGACTTCGC
	R	TCCCAGCCTCCGTTATCC
Mouse Bcl2l1	F	AACAATGCAGCAGCCGAGAGC
	R	GCAGAACCACACCAGCCACAG
Mouse Mcl-1	F	GGAGGAAGAGGACGACCTATACCG
	R	GAAGGCCGTCTCGTGGTTGC
Mouse Bcl2l2	F	ACCTGGCCGCTCAGCTACAC
	R	CCACCATCCAATCCTGCACTTGTC
Mouse Bad	F	TGAGCCGAGTGAGCAGGAAGAC
	R	CGCCTCCATGATGACTGTTGGTG
Mouse Caspase3	F	TGGACTCTGGGATCTATC
	R	TACCAGAGCGAGATGAC
Mouse Caspase7	F	GGGAAAGATGGCGTGACA
	R	AAGGGTGGTCAACGGCTG
Mouse Caspase9	F	TCTGGGACGCTCTGCTGA
	R	CTGCTTGCCGACCGCTTT
CRISPR/Cas9-Bak-Target-1	F	CACCGCAAGTTGTCCATCTCGGGGT
	R	AAACACCCCGAGATGGACAACTTGC
CRISPR/Cas9-Bak-Target-2	F	CACCGTCTTCACCAAGATCGCCTCC
	R	AAACGGAGGCGATCTTGGTGAAGAC
CRISPR/Cas9-Bax-Target-1	F	CACCGCCAGTTCATCTCCAATTCGC
	R	AAACGCGAATTGGAGATGAACTGGC
CRISPR/Cas9-Bax-Target-2	F	CACCGCCCCCCGAGAGGTCTTCTTC
	R	AAACGAAGAAGACCTCTCGGGGGGC
pCI-PP1β	F	CCGCTCGAGATGGCGGACGG
	R	ACGCGTCGACTCACCTTTTCTTC

3. Appendix 3

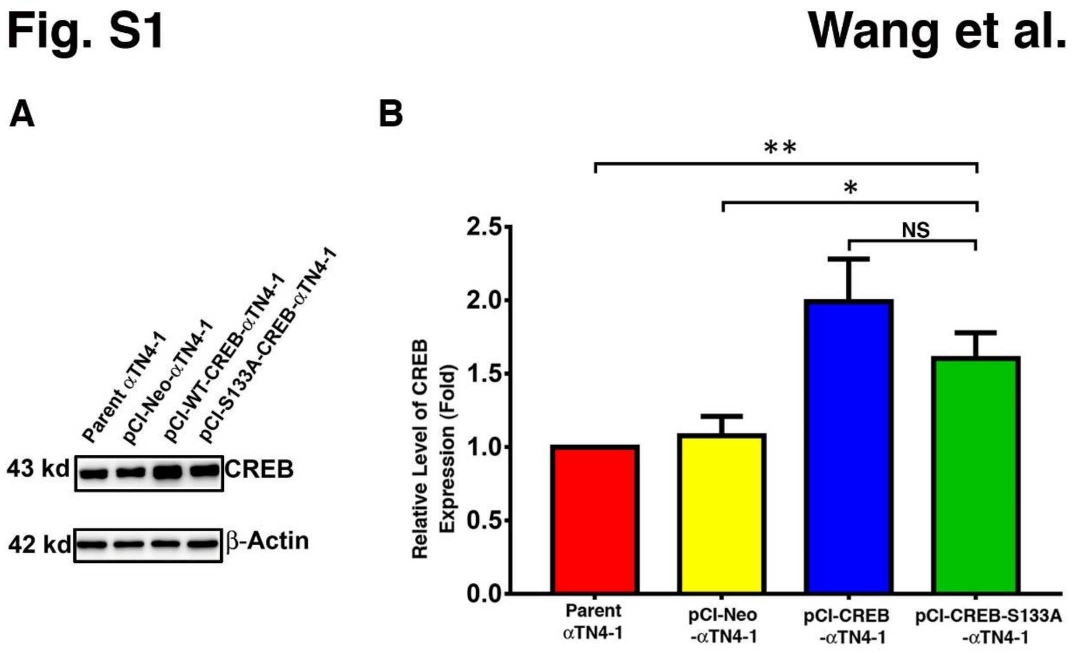

Figure S1. Establishment of αTN4-1 stable cell lines without (Parent) or with expression of the empty vector, exogenous wild type CREB (WT-CREB), or S133A mutant CREB (S133A-CREB). **A.** Western blot analysis of the CREB levels in parent αTN4-1, pCI-Neo-αTN4-1, pCI-CREB-αTN4-1 and pCI-S133A-CREB-αTN4-1 cells. **B**. Quantification of the western blot results in (A). NS, not significant; $*p < 0.05$; $**p<0.01$.

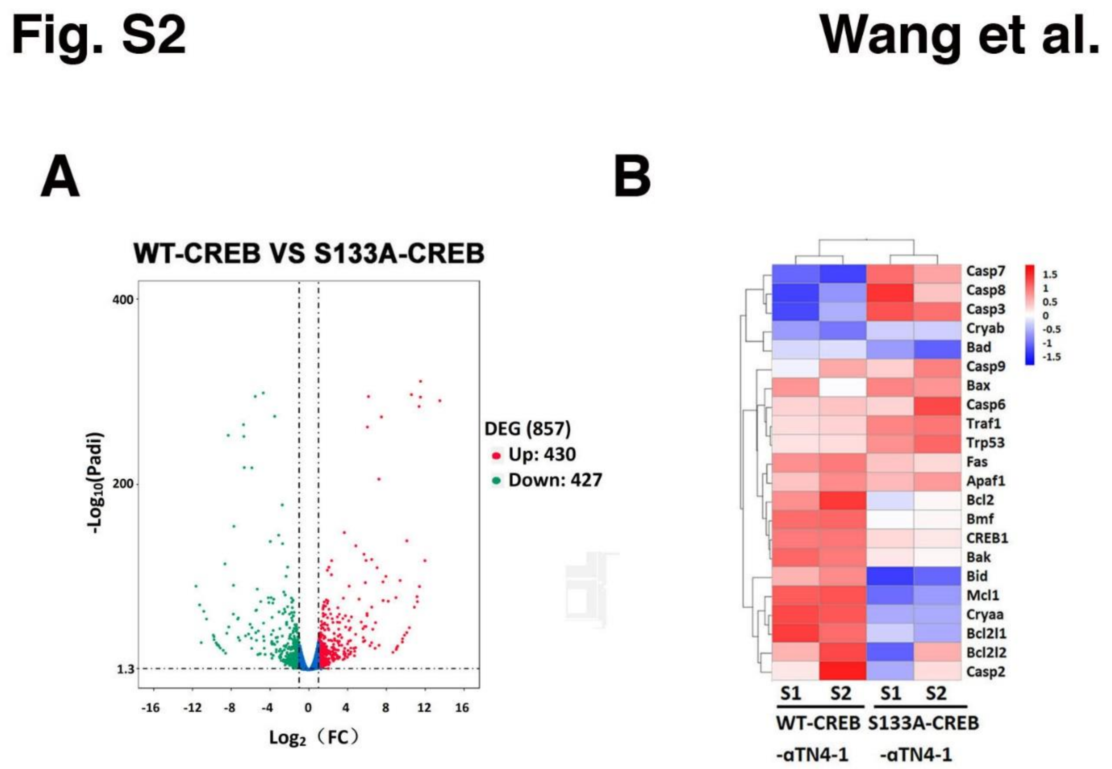

FIGURE S2. Transcriptome analysis of pCI-CREB-αTN4-1 and pCI-S133A-CREB-αTN4-1 cells. **A & B.** pCI-CREB-αTN4-1 and pCI-S133A-CREB-αTN4-1 cells were grown to 95% confluence and then harvested to isolate total RNA for RNAseq analysis. Expression of gene expression patterns between WT-CREB- and S133A-CREB-transfected cells were compared. Compared to WT-CREB-, expression of the exogenous S133A-CREB caused changes in the expression patterns of 857 genes, 430 genes were up-regulated and 427 genes were downregulated (**A**). **B.** Hierarchical cluster heatmap analysis of apoptosis-associated genes.

Fig. S3 **Wang et al.**

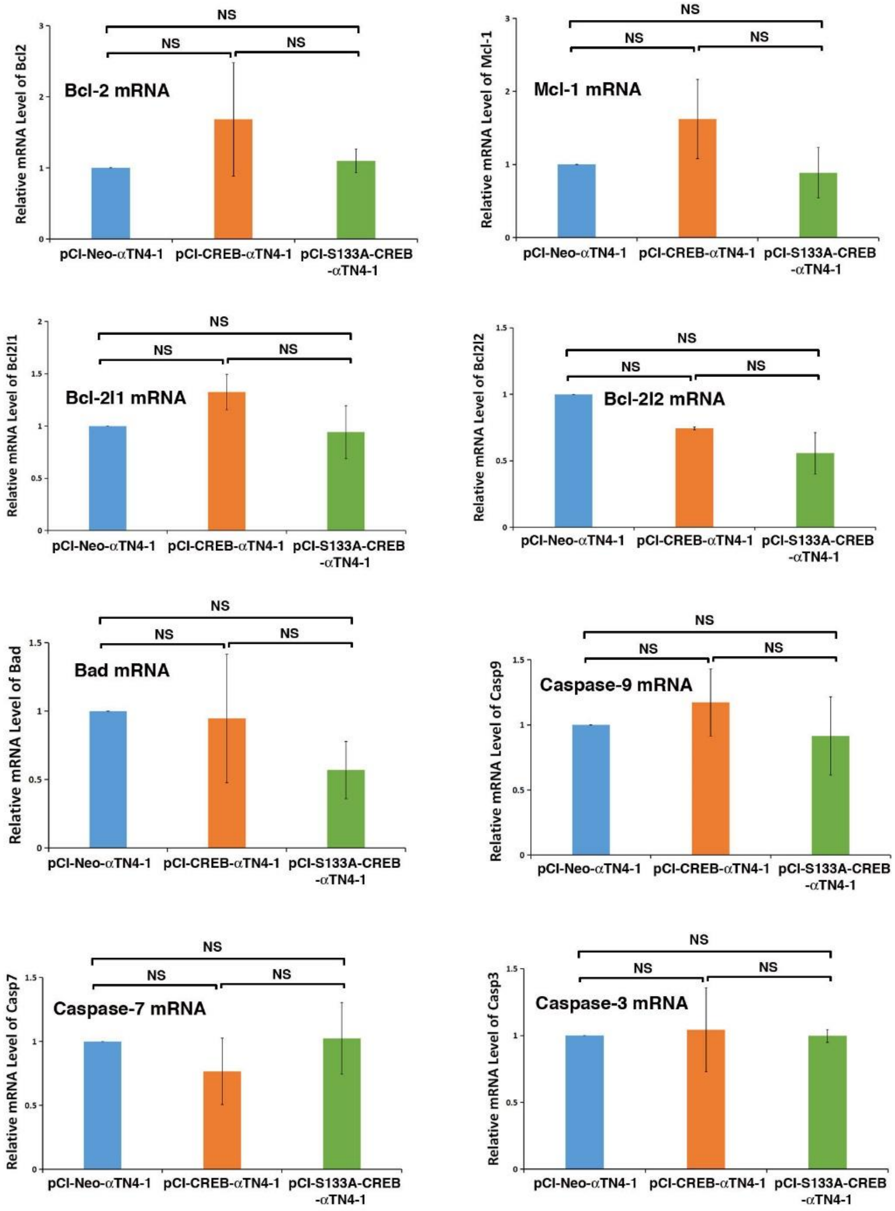

FIGURE S3. qRT-PCR analysis to verify the expression levels of the selected apoptosis-related genes coding for Bcl-2, Mcl-1, Bcl2l1, Bcl2l2, Bad, Caspase-9, Caspase-7 and Caspase-3. No significant changes were observed for these genes. NS: not significant.

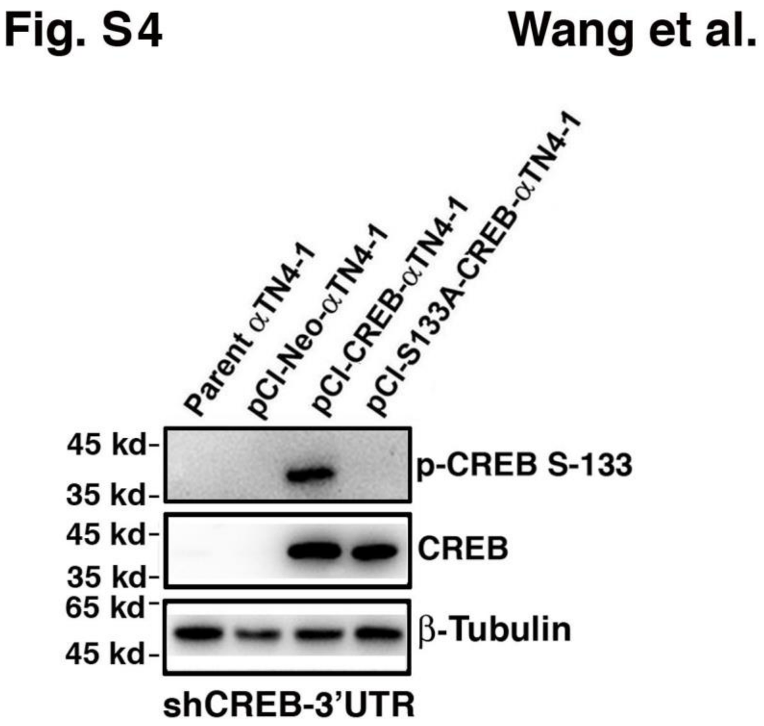

FIGURE S4. Establishment of the endogenous CREB knockdown cells: αTN4-1, pCI-Neo-αTN4-1, pCI-CREB-αTN4-1, pCI-S133A-CREB-αTN4-1 cells. Western blot analysis of CREB in αTN4-1, pCI-Neo-αTN4-1, pCI-CREB-αTN4-1 and pCI-S133A-CREB-αTN4-1 cells with the endogenous CREB knocked down using CREB shRNA targeting to the 3′UTR region.

Fig. S5 Wang et al.

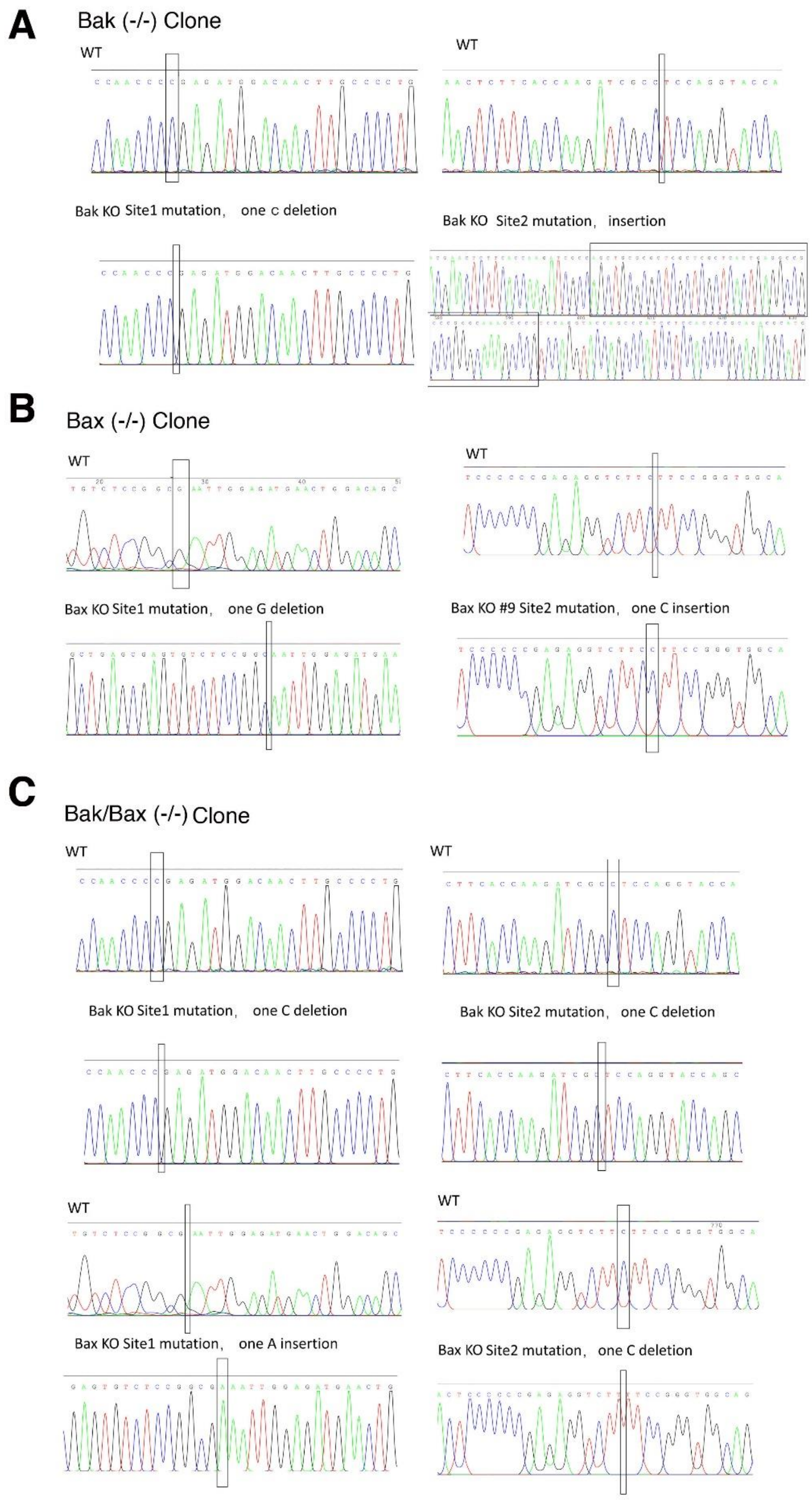

FIGURE S5. DNA sequencing to further verify the Bak (-/-) and Bax (-/-) single knockouts as well as Bak/Bax (-/-) double knockout stable cell lines. To knockout Bak, Bax as well as Bak/Bax in pCI-CREB-αTN4-1 cells, we established the CRISPR/Cas9-vectors targeting two sites for both Bak and Bax genes. **A.** Compared to WT, two mutations have been created in Bak (-/-) clone. In the first targeting site, a cytosine was deleted and in second targeting site, 25 bases were inserted. **B.** For the Bax(-/-) clone, a guanine was deleted in the first targeting site and a cytosine was inserted in the second targeting site. **C.** In the Bak/Bax double knockout clone, there was a cytosine deletion in both first and second target site in Bak (-/-)gene; and an adenine insertion plus a cytosine deletion has been detected in the first and second targeting sites of the Bax gene.

Fig. S6 **Wang et al.**

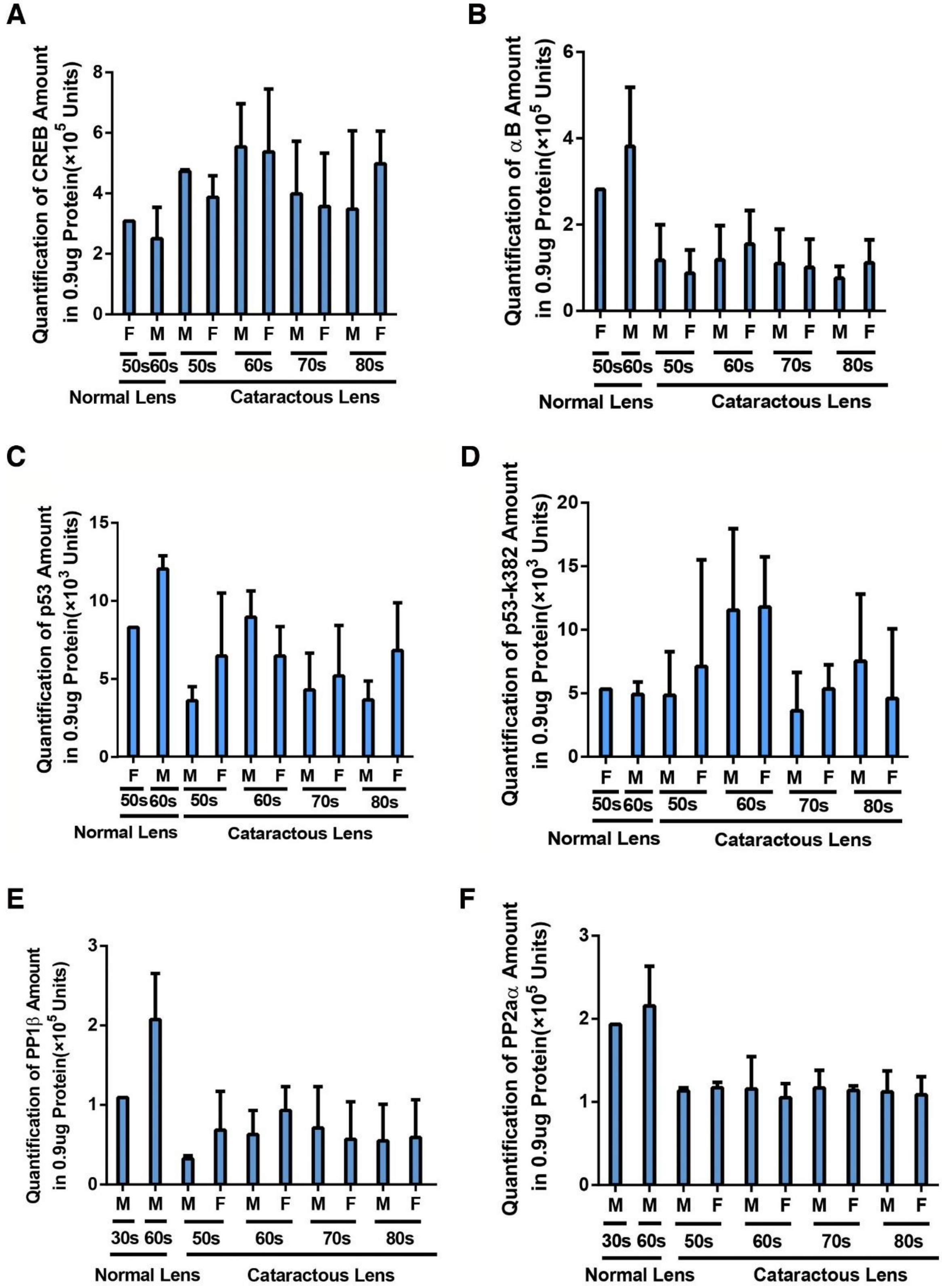

FIGURE S6. Quantification results show gender difference of CREB (**A**), αB-crystallin (**B**), p53 (**C**), p53-K382 (**D**), PP1β (**E**) and PP2Aα (**F**) in normal and cataract lenses. Each bar represents an average of 6 to 10 samples for cataract lenses but 1 to 3 samples for normal human lenses. AWI was performed on a Wes (ProteinSimple) as described recently (Dahl *et al.* 2016; Liu et al., 2020). Briefly, each sample was loaded with 0.9 μg total protein and then analyzed with the Size Separation Master Kit and Split Buffer (12-230 kDa) according to the manufacturer's standard instruction using anti-CREB, anti-αB-crystallin, anti-p53, anti- p53-K382, anti-PP1β and anti-PP2Aα antibodies (for antibody information, see Experimental Procedures) with a dilution factor indicated in the Experimental Procedures. The Compass software (Protein Simple, version 4.1.5) was used to program the Wes and for quantification.

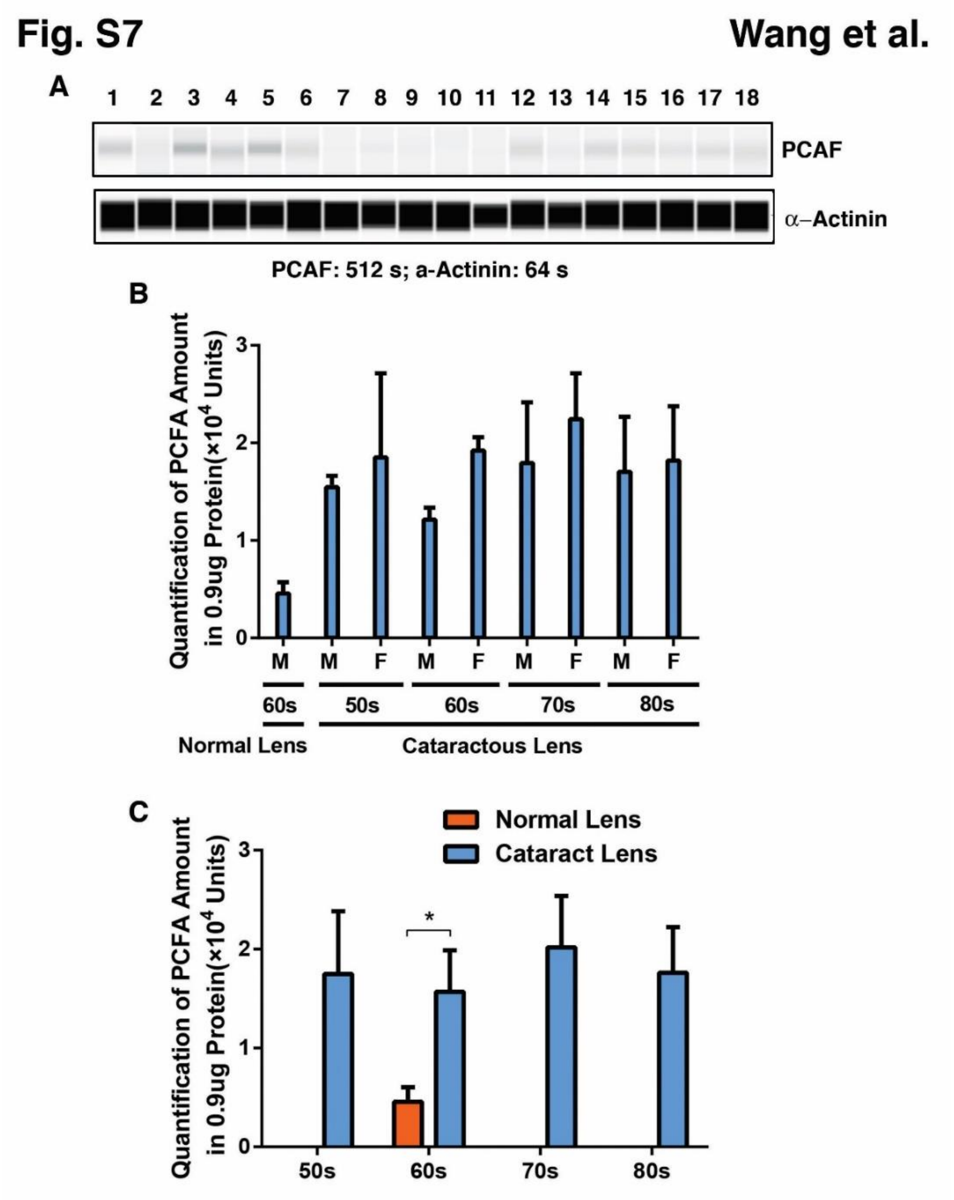

FIGURE S7. The automated Western immunoblot (AWI) analysis of Pcaf in normal and cataractous lenses of different age groups. **A.** Output Western blot style data of Pcaf with exposure time indicated. **B.** Quantification data derived from the software-calculated average of seven exposures (1–512 s) show gender difference. Each bar represents an average of 6 to 10 samples for cataract lenses but 1 to 3 samples for normal human lenses. **C.** Quantification results show gender difference. $^*p < 0.05$.

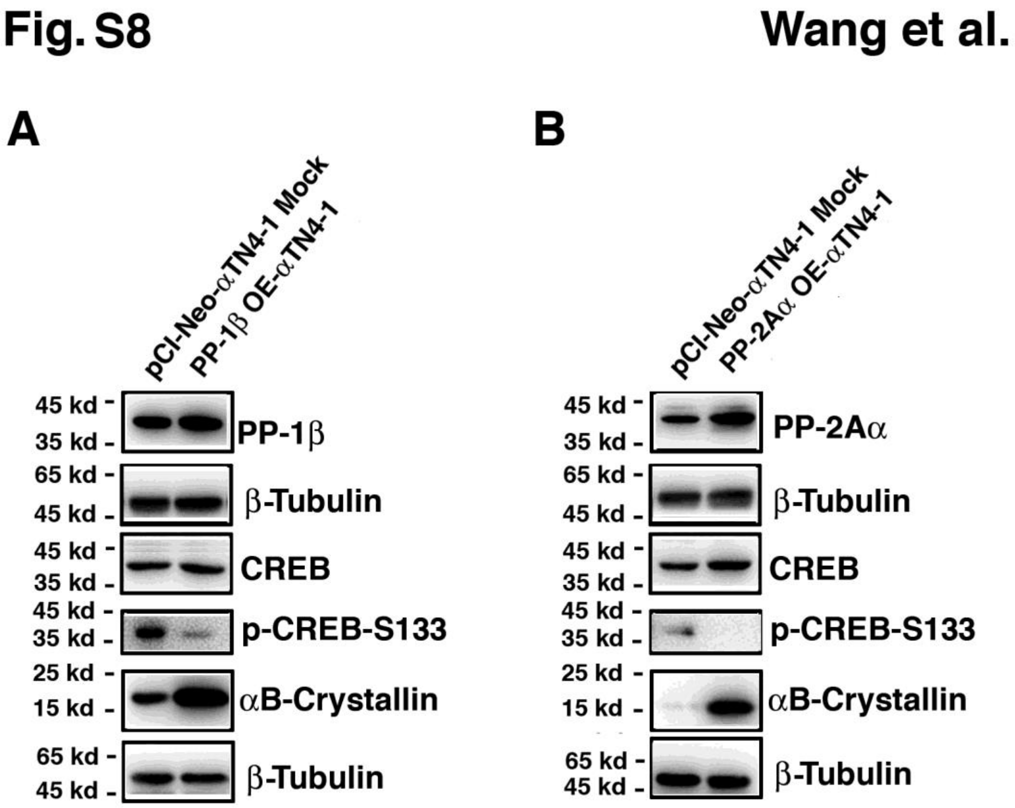

FIGURE S8. Western blot analysis of the effects of PP1β and PP2Aα overexpression on CREB, its phosphorylation status and the expression change of the downstream target, αB-crystallin gene in αTN4-1 cells.
